# The Encapsulation of Illite Powders with Al_2_(SO_4_)_3_·18H_2_O and Hydrophilic Copolymers: Accelerating and Toughening Cement Hydration Through the Proliferation of 54CaO·MgO·Al_2_O_3_·16SiO_2_ Clinker

**DOI:** 10.3390/nano15040283

**Published:** 2025-02-13

**Authors:** Zhiyuan Song, Sidra Chaudhary, Zainab Bibi, Yong Wu, Qinxiang Jia, Xiaoyong Li, Yang Sun

**Affiliations:** 1 Department of Applied Chemistry, School of Chemistry, Xi’an Jiaotong University, No. 28, Xianning West Road, Xi’an 710049, China; 13038012682@163.com (Z.S.); sidra-ch576@stu.xjtu.edu.cn (S.C.); zainabbibi55@stu.xjtu.edu.cn (Z.B.); specwy@mail.xjtu.edu.cn (Y.W.); qinxiangjia1984@mail.xjtu.edu.cn (Q.J.); lixy6658@xjtu.edu.cn (X.L.); 2 Shanxi Jiawei New Material Co., Ltd., Taijia Village, Jiedian Town, Wanrong County, Yuncheng 044200, China; 3 Xi’an Biomass Green Catalysis and Advanced Valorization International Science and Technology Cooperation Base, No. 28, Xianning West Road, Xi’an 710049, China

**Keywords:** illite powders, hydrophilic copolymer, cement, admixture, flexural strength

## Abstract

Two hydrophilic copolymers containing functional groups such as carboxyl, amido, and sulfonic acid are synthesized using ammonium persulfate-catalyzed free radical polymerization in water. Aluminum sulfate is then introduced, resulting in two polymer complexes that exhibit reduced cement setting times (initial, 1.16–2.44 min; final, 2.02–3.14 min) and improved compressive (24 h, 5.81–7.25 MPa) and flexural (24 h, 2.80–2.99 MPa) strengths compared to pure aluminum sulfate-facilitated cementing (initial, 19.11 min; final, 37.05 min; compressive, 24 h, 5.51 MPa; flexural, 24 h, 2.56 MPa). Following this, ball-milled illite powder is added, and the resulting admixtures further display slightly prolonged setting times (initial, 2.35–2.99 vs. 1.16–2.44 min; final, 3.98–4.35 vs. 2.02–3.14 min), along with comparable compressive strengths (5.85–7.11 vs. 5.81–7.25 MPa) and enhanced flexural strengths (3.92–5.83 vs. 2.80–2.99 MPa). Notably, a unique adhesive pozzolanic clinker, Ca_54_MgAl_2_Si_16_O_90_ (54CaO·MgO·Al_2_O_3_·16SiO_2_), emerges in the presence of illite-based admixtures, contributing to the mechanical strength development of the hydrated mortars. Although illite itself is hydrophobic, the coating of ball-milled illite powder with aluminum sulfate and copolymers facilitates its dispersion into the gaps and pores of the cement matrix during setting, thereby increasing the flexural strength. This work presents an interesting approach to utilizing illite materials in cement applications, which is significant for reducing CO_2_ emissions during cement production and use.

## 1. Introduction

Urbanization and the associated construction industry are significant consumers of cement. The production of cement involves the decarbonation of limestone and the combustion of organic materials, which not only requires substantial amounts of electrical energy but also releases considerable quantities of CO_2_, exacerbating the greenhouse effect [1]. A highly promising and effective solution to this challenge is the use of supplementary cementitious materials (SCMs) to partially substitute for cement.

In practice, various materials, including fly ash (the most commonly used SCM) [2,3], silica [4], sludge [5], Granulated Blast Furnace Slag (GBFS) [6], pozzolans [7], tailings [8], and Coal Gangue (CG) [9], have been progressively developed as SCMs. The use of natural clay materials in the cement and concrete industries has also attracted significant and sustained interest from both academic and industrial sectors.

Clay materials exhibit excellent resistance to mechanical crushing, as well as to acidic and alkaline environments, high temperatures, and freezing conditions [10]. Clays are typically nontoxic, odorless, and tasteless, significantly reducing the risk of environmental pollution [10]. Moreover, the abundant reserves of clay minerals worldwide can optimize the production costs of cement materials, contributing to future architectural advancements [10].

The clays commonly utilized in construction materials primarily include kaolinite, smectite, and illite. Kaolinite is typically classified as a 1:1-type phyllosilicate, based on the ratio of its layered structures, which consist of tetrahedral (silicon) and octahedral (aluminum) sheets [10]. However, both raw kaolinite rocks and their mechanically milled powders are rarely employed as SCMs. Calcined kaolinite, known as metakaolin (MK), exhibits significant pozzolanic activity [11]. During calcination (500 °C or higher), kaolinite (Al_2_O_3_·2SiO_2_·2H_2_O, AS_2_H_2_) undergoes dehydration and dehydroxylation, resulting in the formation of MK (Al_2_O_3_·2SiO_2_, AS_2_) [12].

In cement hydration, MK reacts with portlandite (Ca(OH)_2_, CH) generated during hydration to produce new hydration products, including calcium aluminosilicate hydrate gel (CaO·Al_2_O_3_·SiO_2_·H_2_O, C–A–S–H) [13]. This gel plays a crucial role in refining the pore structure and densifying the overall structure of the hydrated matrix, resulting in enhanced compressive strength and, in certain cases, improved durability [13].

However, there are significant shortcomings associated with the application of MK as a SCM. First, the availability of pure kaolinite clays worldwide is relatively limited, and their market price has increased due to demand from the paper and ceramics industries [14]. Second, the more readily accessible kaolinitic clays, referred to as “low-grade kaolinitic clays”, typically consist of a mixture of kaolinite, montmorillonite, and illite. These composites exhibit varying pozzolanic activity, complicating their use considerably [11].

Smectite clays are classified as 2:1-type phyllosilicates, characterized by tetrahedral SiO_4_ and octahedral AlO_6_ sheets, with montmorillonite (MM) being the most prominent representative [10]. The introduction of MM can significantly impact the engineering performance of CaO-rich binder systems, such as Ordinary Portland Cement (OPC). However, the incorporation of raw MM typically leads to reductions in bleeding, evaporation, plastic settlement, consistency, and fluidity in fresh cement pastes and mortars as the dosage of MM increases [15].

The first method of activating MM to produce suitable SCMs was calcination, which saw widespread application in large-scale construction during the 1960s [10]. The pozzolanic reactivity of MM can be demonstrated through calcination, which involves three continuous processes: dehydration (100–200 °C), dihydroxylation (400–800 °C), and amorphization (750–950 °C) [16]. The mechanical activation of MM has also garnered increasing attention as a promising approach to enhance the chemical reactivity of clays by decreasing particle size, creating dislocations, and introducing defects in activated MM, thereby creating new types of SCMs [17].

**Figure 1:**
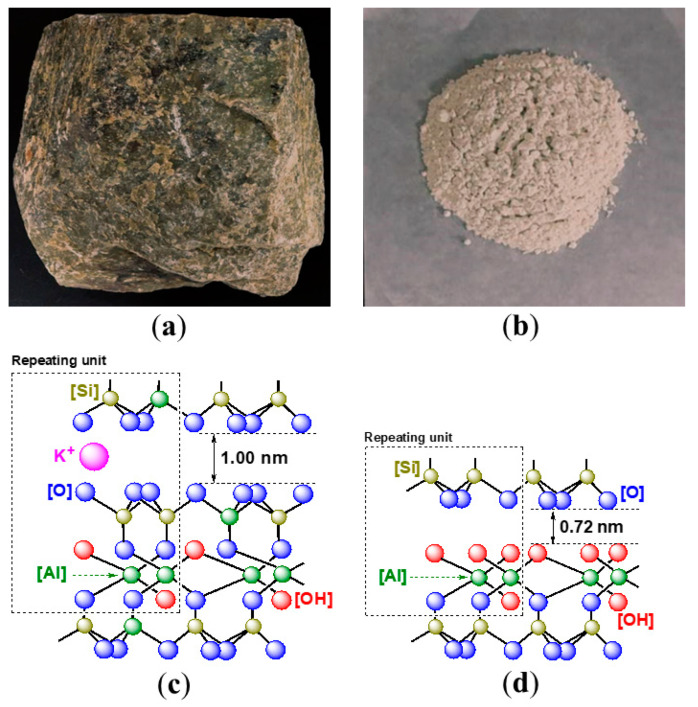
Appearances and structures of clays: (**a**) raw illite rock, (**b**) ball-milled illite, (**c**) structure of illite, (**d**) structure of kaolinite.

Illite is an abundant clay mineral found worldwide. Raw illite rocks exhibit a cyan color (Figure 1a), while their ball-milled powders remain white (Figure 1b). From a structural perspective, illite clays are classified as 2:1-type phyllosilicates too, characterized by a flaky crystal structure composed of two tetrahedral (silica) sheets sandwiching one octahedral (aluminum) sheet (Figure 1c) [10]. Additionally, magnesium ions can substitute for aluminum ions at certain sites within the octahedral sheet [10].

The hydroxyl units of illite are encapsulated by octahedral (aluminum) sheets, which distinguishes them from those found in kaolinite (Figure 1c vs. Figure 1d). Consequently, to fully realize the pozzolanic properties of illite, calcination at 950 °C is required, significantly higher than the 500 °C needed for the activation of kaolinite. This high-temperature treatment results in the formation of amorphous aluminosilicate following the dehydroxylation and structural collapse of illite [16].

In practice, the cement industry primarily focuses on utilizing common clays, such as illite, for pozzolan production due to their availability and low cost. However, there are several drawbacks associated with the use of illite. Firstly, the thermal activation process not only consumes a significant amount of electricity but also results in substantial emissions of CO_2_ and other pollutants.

Secondly, due to the differences in layer structures, the solubilities of aluminum and silicon ions in MK can exceed 10 mmol L^−1^, whereas those in illite are considerably lower [10]. This disparity slows down the hydration of cement and hinders the development of mechanical strength, particularly compressive strength, when using illite. Additionally, there is a notable lack of research on the mechano-activation of illite and its corresponding applications.

Cement accelerators are crucial in the construction of tunnels, bridges, and other specialized structures that require the rapid setting and high early strength development of cement. Without accelerators, the integrity of the entire structure could be compromised due to the weight of concrete components. Currently, aluminum sulfate (Al_2_(SO_4_)_3_·18H_2_O, AS) is the leading accelerator available on the market and is widely used in construction [18]. This is largely attributed to its high efficiency in accelerating the setting process, enhancing the early mechanical strength of mortar, low resilience, environmental friendliness, and safety for human health [19].

In its mechanism, during the hydration of OPC, which is rich in calcium oxide (CaO), the pH of the hydrated mortar gradually increases due to the hydrolysis of CaO into CH. When AS is introduced, Al^3+^ ions are readily hydrolyzed to form [Al(OH)_4_]^−^, which reacts immediately with CaO to produce tricalcium aluminate (C_3_A, 3CaO·Al_2_O_3_) [20].

C_3_A subsequently combines with gypsum (CaSO_4_·2H_2_O), a key component of raw cement, resulting in the formation of ettringite (calcium sulfoaluminate hydrate, 3CaO·Al_2_O_3_·3CaSO_4_·32H_2_O, AFt). This compound acts as a powerful rod-like clinker within the three-dimensional cement network, working in conjunction with layered C–S–H gel (hydrated tricalcium silicate, 3CaO·SiO_2_·3H_2_O) [20]. Together, they accelerate cement hydration and significantly enhance the early mechanical strength of the mortar.

However, the low water solubility of AS (36.5 g AS per 100 g distilled H_2_O at 20 °C) [21] poses a challenge when using AS as the sole accelerator, as it can lead to the immediate precipitation or hydrolysis of Al^3+^ into unreactive Al(OH)_3_ or Al_2_O_3_ in water, thereby inhibiting hydration [20]. Consequently, stabilizing Al^3+^ in an aqueous environment is crucial for maintaining the efficacy of AS-based accelerators [20]. One potential solution to this issue is the introduction of hydrophilic coordinating ligands, which could effectively enhance the stability of Al^3+^.

The hydration reaction of cement also creates a significant number of capillary pores in the mortar, resulting in reduced inherent mechanical strength [22]. This deficiency could be mitigated by incorporating illite nanoparticles, which may enhance the mechanical strength of the mortar. However, due to the hydrophobic nature of illite’s surface, the filling of these pores may be incomplete, and the adhesive effects may not be sufficient to sustain a high level of mechanical strength in the hydrated product. Therefore, the hydrophilic coating on the illite dopant appears to be a promising approach to address this issue.

On the other hand, the use of organic polymers as functional admixtures in cementing has made significant progress. For instance, polycarboxylate ether (PCE) is employed as a superplasticizer, generating negative charges at high pH levels. This allows PCE to adsorb onto positively charged cement particles, preventing their agglomeration, eliminating micropores during the cementing process, and enhancing the mechanical strength of the mortar, while also reducing water consumption [23]. However, the rheological properties of PCE need further improvement to facilitate the more rapid setting of cement.

Considering the advantages and limitations of the current polymer admixtures used in cementing, it is crucial to explore new organic coatings that can enhance the functionality of illite and AS particles during cement setting. Overall, the design and application of highly hydrophilic copolymers with versatile functional groups appear to be a more viable option compared to other known homopolymers.

The selection of copolymer monomers, then, warrants attention. For instance, acrylic acid (AA) features a carboxyl group that can effectively coordinate with Ca^2+^ and Al^3+^ ions while readily releasing H^+^. This property may help prevent the agglomeration of cement particles and mitigate the ineffective hydrolysis of AS [24]. Furthermore, maleic acid (MA) contains two carboxyl groups, and its incorporation into the copolymer could further enhance the benefits associated with AA.

Alternatively, itaconic acid (IA) also possesses two carboxyl groups, like MA; however, one of IA’s carboxyl groups is linked to a C=C bond via a methylene group. This structural difference may improve its lipophilicity to some extent, potentially enhancing the plasticity of the mortar [25]. In addition, the inclusion of 2-acrylamido-2-methylpropane sulfonic acid (AMPS) may augment the rheological properties of the mortar [25], which could have specific effects on cement hydration.

This study aims to achieve the following: (1) accelerate the cementing process by inhibiting the ineffective hydrolysis of AS through the introduction of a hydrophilic coordinating copolymer, and (2) enhance the mechanical strength of hydrated mortar by filling the pores and crevices of the cement matrix with ball-milled illite powder coated with the same copolymer. Ultimately, this research seeks to reduce the amount of cement used in construction, thereby decreasing CO_2_ emissions into the atmosphere. Initially, natural illite rock is activated through ball milling to produce powders. Concurrently, two copolymers, *p*(AA–*co*–MA–*co*–AMPS) and *p*(AA–*co*–IA–*co*–AMPS), are synthesized via (NH_4_)_2_S_2_O_8_-initiated aqueous free radical polymerization. The resulting copolymers are then combined with AS in an aqueous solution, along with the addition of powdered illite, to create a series of admixtures.

These admixtures are subsequently introduced into the hydration process of OPC. This study not only evaluates the setting times of the cement pastes and the mechanical strengths of the mortars, but also examines the effects of various parameters, including admixture type and dosage, on cement hydration. Additionally, both microstructural and morphological analyses are conducted to identify potential new hydrated products and elucidate the underlying mechanisms. This research primarily seeks to improve the use of common clays in the construction industry, while also contributing to a reduction in CO_2_ emissions.

## 2. Experimental

### 2.1. Raw Materials

The monomers used for the synthesis of two copolymers included acrylic acid (AA, 99%), maleic acid (MA, 99%), itaconic acid (IA, 99%), and 2-acrylamido-2-methylpropane sulfonic acid (AMPS, 98%). The copolymerization initiator, ammonium persulfate ((NH_4_)_2_S_2_O_8_, 98%), along with these monomers, was purchased from Shanghai Aladdin Biochemical Technology Co., Ltd. (Shanghai, China). The solvent for copolymerization, *n*-BuOH (*n*-butanol, 99%), as well as aluminum sulfate (Al_2_(SO_4_)_3_·18H_2_O, AS, 99%), were sourced from Shanghai Macklin Biochemical Technology Co., Ltd. (Shanghai, China).

The reagents used for determining the monomer conversions of the synthesized copolymers included potassium bromate (KBrO_3_, 99.5%), potassium bromide (KBr, 99%), mercuric sulfate (HgSO_4_, 99%), concentrated sulfuric acid (H_2_SO_4_, 98%), sodium chloride (NaCl, 99.5%), potassium iodide (KI, 99%), and sodium thiosulfate (Na_2_S_2_O_3_, 99%). All these chemicals were procured from Alfa Aesar (Haverhill, MA, USA) and Thermo Fisher Scientific (China) Co., Ltd. (Shanghai, China).

The raw illite rocks (Figure 1a) were obtained from Hua’an County Jiulonghu Sandstone Co., Ltd. (Zhangzhou, China). Cement (P·O 42.5) was supplied by the China National Academy of Building Materials Science Co., Ltd. (Beijing, China) The Chinese ISO standard sand, produced in accordance with GB/T 17671-2021 [26], was purchased from Xiamen ISO Standard Sand Co., Ltd., Xiamen, China. Distilled water was prepared in our laboratory.

### 2.2. Instruments and Processes for Mechano-Activation of Illite Rocks

The ball milling of the illite rocks (Figure 1a) was conducted using a YJKS-Speediness Grind Machine from Foshan Tenghao Instrument Technology Co., Ltd. (Foshan, China). This machine features a two-vessel configuration, operates at a voltage of 220 V, and has a power output of 370 W. The milling beads used were made of ZrO_2_ and had a diameter of 18 mm.

The ball milling of illite rocks was conducted for 3 h using distilled water as a grinding aid. Following the milling process, the powders were dried in an oven at 120 °C for 24 h. The dried powders were then sieved through a 300-mesh sieve, and the resulting particles (Figure 1b) were collected for future use.

### 2.3. Synthesis of Admixtures

**Figure 2:**
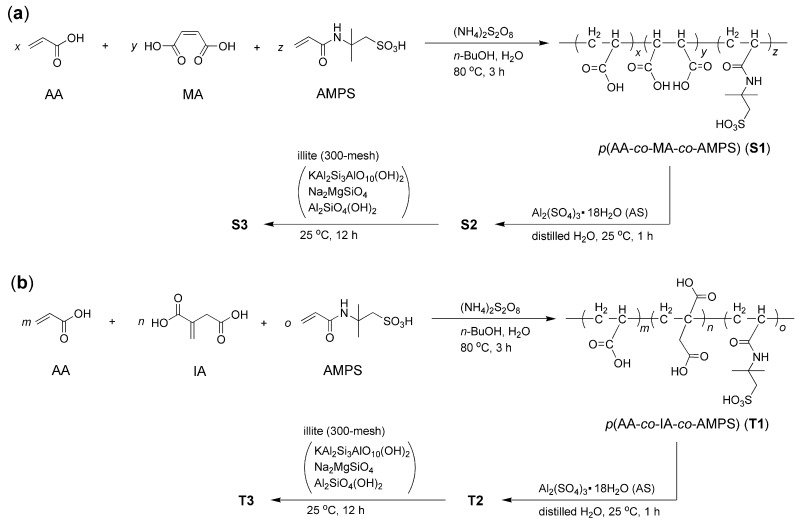
(**a**,**b**) Synthesis of intermediates and illite-based admixtures.

As depicted in Figure 2, a mixture of AA (200 g, 2.76 mol), MA (200 g, 1.72 mol for the synthesis of S1; or IA, 200 g, 1.53 mol for T1), AMPS (200 g, 0.96 mol), and *n*-BuOH (600 mL, 488.88 g, 6.57 mol) was combined into a 2 L three-necked flask featuring a condenser, an addition funnel, and a mechanical stirrer. To this mixture, the solution of (NH_4_)_2_S_2_O_8_ (4 g, 17.2 mmol) dissolved in distilled H_2_O (100 mL) was added slowly through the addition funnel while stirring vigorously at 25 °C within 1 h. The temperature was then gradually increased to 80 °C, and the mixture was stirred continuously at this temperature for 3 h. When cooling to room temperature, the solvent was completely removed by rotary evaporation, resulting in a slightly yellow oil, designated as *p*(AA–*co*–MA–*co*–AMPS) (S1) or *p*(AA–*co*–IA–*co*–AMPS) (T1).

Subsequently, S1 (or T1, 100 g) was combined with AS (350 g) and distilled water (150 mL) in a 1 L three-necked flask with a mechanical stirrer at 25 °C. After vigorous stirring for 1 h, a white mixture was formed and stored for future use, named as S2 (or T2). Next, S2 (or T2, 600 g) was mixed with ball-milled illite powder (35.0 g) in a 1 L round-bottom flask, featuring a mechanical stirrer, at 25 °C. The mixture was stirred vigorously at 25 °C for 12 h, resulting in S3 or T3, respectively.

### 2.4. Measurement of Monomer Conversions of Synthesized Copolymers

**Figure 3:**
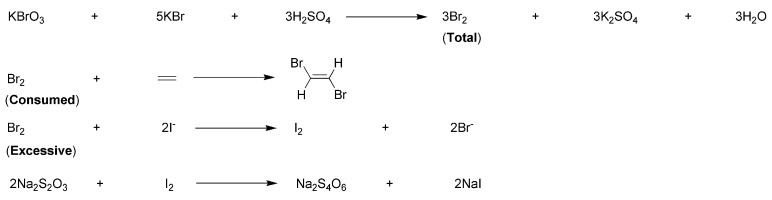
Illustrations of chemical conversions during determination of bromine numbers of synthesized copolymers.

The monomer conversion (*α*, %) of the synthesized copolymer was calculated from the bromine number (*X*, mg g^−1^). The bromine number (*X*, mg g^−1^), which indicates the amount of bromine consumed per gram of synthesized copolymer, was determined in accordance with the Chinese standard GB/T 10535-2014 [27]. In this method, Br_2_ (generated in situ) covalently reacted with the unpolymerized monomers left in the sample. Any excess Br_2_ then reacted with the added KI solution, resulting in the precipitation of I_2_, which was quantified through standard titration with Na_2_S_2_O_3_. The relevant chemical conversions are shown in Figure 3.

In practice, S1 (or T1, 0.5000 g) was dissolved with distilled H_2_O into a 250 mL volumetric flask, to the fixed volume. An aliquot of this solution (25.00 mL) was then moved to an iodine flask (250 mL), to which a combined solution of KBrO_3_ and KBr (10 mL) was added. This mixed solution was made by dissolving KBrO_3_ (5.5 g) and KBr (20.0 g) in distilled H_2_O, bringing the total volume to 1000 mL in a brown volumetric flask. After shaking the mixture for 5 min, 3 mol L^−1^ H_2_SO_4_ solution (20 mL) was added, followed by adding HgSO_4_ solution (5 mL), which was made by dissolving HgSO_4_ (15 g) in concentrated H_2_SO_4_ (14 mL) and diluting it to 475 mL with distilled H_2_O. The resulting solution was thoroughly mixed and then stored at 0–20 °C in a dark place for 30 min.

Next, NaCl solution (15 mL, 116 g L^−1^) and KI solution (10 mL, 100 g L^−1^) were added, and the solution was then shaken thoroughly and stored in a well-lit area for 5 min at room temperature. Following this, distilled water (20 mL) was added. The solution was subsequently titrated with a standard Na_2_S_2_O_3_ solution (0.1 mol L^−1^) until a faint yellow color occurred. At this point, starch indicator solution (1 mL, 10 g L^−1^) was added, and the titration continued until the solution transitioned from blue to colorless.

The bromine number (*X*, mg g^−1^) was then calculated by using Equation (1):

(1) $$X=\frac{c\times (V_{0}-V)\times 0.0799\times 1000}{m}$$

Herein: *X* is bromine number (mg g^−1^); *c* is real concentration of standard Na_2_S_2_O_3_ solution (mol L^−1^); *V*_0_ is consumed volume of standard Na_2_S_2_O_3_ solution in sample blank experiment; *V* is consumed volume of standard Na_2_S_2_O_3_ solution in regular experiment; 0.0799, is bromine (Br) mass (g) derived from consumption of ideal Na_2_S_2_O_3_ solution (1 mL, 1.000 mol L^−1^); and *m* is mass of tested copolymer.

The monomer conversion (*α*, %) of the tested copolymer was calculated by using Equation (2):

(2) $$\alpha =1-\frac{Xm_{0}}{1000\times M\times (m_{1}/M_{1}+m_{2}/M_{2}+m_{3}/M_{3})}$$

Herein: *α* is monomer conversion of tested sample; *X* is bromine number (mg g^−1^); *m*_0_, *m*_1_, *m*_2_, *m*_3_, are masses of all monomers, AA, MA (or IA), AMPS, respectively; and *M*, *M*_1_, *M*_2_, *M*_3_, are molecular weights of Br, AA, MA (or IA), AMPS, respectively.

### 2.5. Instruments and Processes for Measuring Setting Times of Cement Pastes

The IST (initial setting time) and FST (final setting time) of the paste were measured by using a Vicat apparatus following the combination and mixing of the cement and admixture in an NJ-160A cement paste mixer. Both the Vicat apparatus and the NJ-160A mixer were manufactured by Wuxi Xiyi Building Material Instrument Factory (Wuxi, China).

According to the Chinese standard JC 477-2005 [28], the IST and FST of cement paste were tested as follows: initially, the cement (400 g) was mixed with distilled H_2_O (148 g for an admixture dosage of 6 wt.% over cement, 144 g for a 7 wt.% dosage, and 140 g for an 8 wt.% dosage). This mixture was stirred at low speed for 30 s. Next, the admixture was added in different quantities: 24 g for the 6 wt.% dosage, 28 g for the 7 wt.% dosage, and 32 g for the 8 wt.% dosage. The resulting mixture was stirred at low speed for 5 s, followed by high-speed stirring for 15 s. The prepared cement paste was then immediately poured into a round mold, compacted, and vibrated lightly. Finally, the surface of the paste was smoothed using a scraper.

Both the IST and FST were reported every 10 s using the Vicat apparatus, meaning penetrating the cement paste with a needle of fixed cross-section under a constant force. The IST was tested as the time elapsed from the moment the needle was released in free fall until it reached a depth of 4 ± 1 mm from the bottom of the paste. The FST was tested as the duration between the end of the IST and the moment when the needle could not penetrate the paste any more.

### 2.6. Instruments and Processes for Measuring Mechanical Strengths of Cement Mortars

The cement mortar mixer was JJ-5, made by Wuxi Xiyi Building Material Instrument Factory in Wuxi City, China. The compressive and flexural strengths of the mortar were evaluated using a fully automatic anti-folding and compression testing machine, the WAY-300B. This machine features an advanced control system, the EHC-2300, with a maximum capacity of 300 kN and a pressing speed of 48 N s^−1^. The mortar specimens were cured in a numerical control standard cement conservation box, the HBY-40B, which was maintained at 20 °C and 90% humidity. All testing instruments were also produced by the Wuxi Xiyi Building Material Instrument Factory.

In practice, the compressive and flexural strengths of the cement mortars were tested according to the Chinese standard JC 477–2005 [28]. Initially, the cement (900 g) was mixed with distilled H_2_O (468 g), for an admixture dosage of 6 wt.% over cement, 459 g for 7 wt.%, and 450 g for 8 wt.%, in a mixing bowl. This mixture was stirred at low speed for 30 s using the cement mortar mixer. Following this, an additional 30 s of low-speed stirring was performed before gradually adding Chinese ISO standard sand (1350 g).

The mixture was then mechanically stirred at high speed for 30 s, then left to rest for 90 s, and stirred again at high speed for 30 s. Soon after stirring, the admixture was added in different amounts: 54 g for a 6 wt.% dosage, 63 g for a 7 wt.% dosage, and 72 g for an 8 wt.% dosage. The mixture was stirred at low speed for 5 s, followed by high-speed stirring for 15 s.

The cement mortar was subsequently promptly transferred into a mold of size 40 mm × 40 mm × 160 mm (trial mold for soft scouring) and stored in a cement conservation box at 20 °C under 90% humidity for predetermined incubation periods of 6 h, 24 h, and 28 days.

### 2.7. Instruments for Microstructural and Morphological Characterizations

FT-IR spectra were taken using a VERTEX 70 instrument, made by Bruker (Berlin, Germany), at 400 to 4000 cm^−1^. X-ray photoelectron spectroscopy (XPS) measurements were performed on a Kratos Axis Ultra DLD (Kratos Co., Ltd., Manchester, UK), using monochromatic Al-Kα X-rays (1486.6 eV) as the excitation source. The binding energy scale was calibrated by setting the C 1s peak at 284.8 eV (sp^3^ hybridized, saturated carbon) as the reference. Peak fitting was carried out by using a Gaussian–Lorentz (G/L) product function, applying a 30% Lorentzian ratio.

Wide-angle (2*θ* = 10–80°) X-ray diffraction (XRD) patterns of the powdered samples were measured by using a Philips X’Pert Pro diffractometer (PANalytical B.V. Co., Ltd., Almelo, The Netherlands), using Cu-Kα radiation (λ = 1.5418 Å) with a scan rate of 0.05° s^−1^.

Thermogravimetric analysis, including both TGA (thermogravimetric analysis) and DTG (derivative thermogravimetry) of the prepared samples, was conducted on a METTLER instrument manufactured by METTLER TOLEDO, Zurich, Switzerland, utilizing a heating rate of 10 °C min^−1^. Scanning electron microscopy (SEM) images were acquired using the GeminiSEM 500 instrument, manufactured by Carl Zeiss (Shanghai) Management Co., Ltd., Shanghai, China.

The measurement of aqueous particle size and ζ (zeta) potential was performed using a Zetasizer Nano ZS90 spectrometer from Malvern (Malvern, UK). Inductively Coupled Plasma Optical Emission Spectroscopy (ICP-OES) was performed on an Agilent 5110, manufactured by Agilent Technologies (Santa Clara, CA, USA), with a pump rate of 60 rpm, while the plasma gas flow was 12.0 L min^−1^, nebulizer flow was 0.70 L min^−1^, stable time was 20 s, auxiliary gas flow was 1.0 L min^−1^, reading access time was 5 s, sample flush time was 20 s, and RF power was 1250 W.

The samples used for microstructural and morphological characterizations were obtained by mixing specimens of three parallel experiments.

## 3. Results and Discussion

### 3.1. Characterizations of Admixtures

#### 3.1.1. Characterizations of Copolymers and Al-Containing Polymer Complexes

The synthesis of copolymers (S1 and T1) is illustrated in Figure 2, and the corresponding *X* and *α* values are presented in Table 1. Generally, (NH_4_)_2_S_2_O_8_ serves as an effective initiator for the aqueous free radical polymerization of AA, MA (or IA), and AMPS at a temperature of 80 °C (Figure 2) [29]. Notably, the incorporation of MA results in a higher monomer conversion compared to IA (Table 1). This difference may be attributed to the slightly increased lipophilicity of IA, which is influenced by the presence of a methylene group adjacent to the carboxyl group in its structure (Figure 2b).

**Figure 4:**
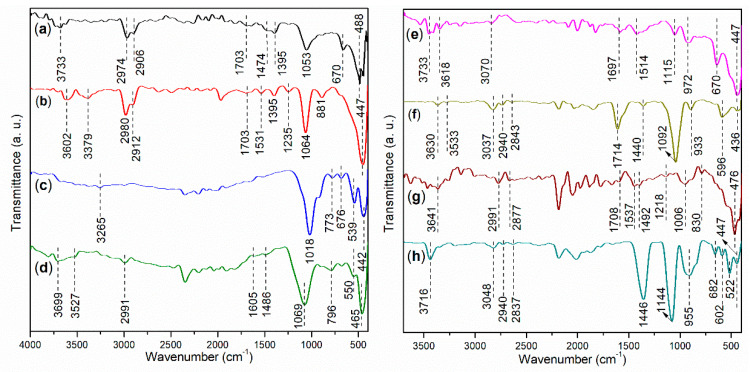
FT-IR spectra of synthesized samples and raw materials: (**a**) S1; (**b**) S2; (**c**) illite; (**d**) S3; (**e**) T1; (**f**) T2; (**g**) T3; (**h**) cement.

The FT-IR spectra of the synthesized copolymers and corresponding Al-containing polymer complexes are presented in Figure 4. Firstly, S1 exhibits a small broad band at 3733 cm^−1^ (Figure 4a), which indicates the O–H stretching vibration of the carboxyl groups from AA and MA, as well as the sulfonic acid group from AMPS (Figure 2a) [30]. Upon coordination with Al^3+^, this band is red-shifted to 3602 cm^−1^ and 3379 cm^−1^ (Figure 4b vs. Figure 4a). Furthermore, the peaks observed at 2974 cm^−1^ and 2906 cm^−1^ in S1 correspond to the anti-symmetric and symmetric stretching of the C–H bonds in the methyl group of the AMPS monomers (Figure 4a) [31], and these peaks are also present in S2, albeit with slight blue shifts (Figure 4b). Additionally, S1 displays a small band at 1703 cm^−1^ (Figure 4a), which suggests the C=O stretching of the carboxyl group [30]. This band is nearly retained in S2 without any shifts (Figure 4b), indicating that the C=O group in S1 may not coordinate with Al^3+^ during the formation of S2.

S1 exhibits two peaks at 1474 cm^−1^ and 1395 cm^−1^ (Figure 4a), both of which correspond to C–C stretching vibrations [30]. The next prominent peak at 1053 cm^−1^ can be attributed to C–O stretching in the carbonyl group (O=C–OH) [30]. Additionally, S1 displays a peak at 670 cm^−1^, which corresponds to the in-plane bending vibrations of C–O bonds [30]. In comparison, S2 shows a similar spectral profile to S1 at 1703–400 cm^−1^ (Figure 4b vs. Figure 4a). However, a distinctive peak at 447 cm^−1^ in S2 can be attributed to the stretching of the Al–O bond (Figure 4b).

A comparison of T1 with T2 reveals a similar trend to that observed between S1 and S2 (Figure 4f vs. Figure 4e and Figure 4b vs. Figure 4a). Notably, the peak at 1697 cm^−1^ in T1, which characterizes the C=O stretching of the carboxyl group, is blue-shifted to 1714 cm^−1^ in T2 (Figure 4f vs. Figure 4e). This shift may suggest that the carboxyl groups are partially polarized towards the polyesters during the coordination with Al^3+^.

#### 3.1.2. Characterizations of Illite and Illite-Based Admixtures

The FT-IR spectrum of illite reveals a small peak at 3265 cm^−1^ (Figure 4c), which likely corresponds to the O–H stretching vibrations of the hydroxyl groups in illite (Figure 1c). The subsequent peaks at 1018 cm^−1^ and 773 cm^−1^ may indicate the anti-symmetric and symmetric Si–O stretching vibrations [32]. A vibration observed at 676 cm^−1^ can be attributed to the in-plane bending vibrations of C–O bonds from the organic residues in illite [30]. Finally, illite displays two sharp peaks with moderate intensities at 539 cm^−1^ and 442 cm^−1^, which are likely indicative of Mg–O and Al–O stretching vibrations.

After the illite is combined with S1 and AS, the resulting admixture, S3, exhibits FT-IR characteristics that reflect the combined effects of both S2 and illite (Figure 4d vs. Figure 4b,c). A comparison of T3 with T2 and illite reveals a similar trend (Figure 4g vs. Figure 4c,f).

**Figure 5:**
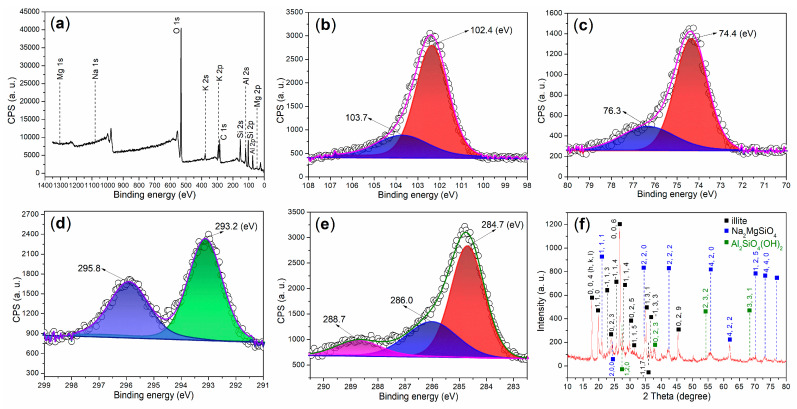
XPS of illite: (**a**) survey scan; (**b**) Si 2p; (**c**) Al 2p; (**d**) K 2p; (**e**) C 1s; XRD of illite: (**f**) wide-angle (2*θ* = 10–80°) XRD.

It is both interesting and essential to investigate the surface and bulk compositions, as well as the elemental valence of illite, using XPS and XRD (Table 2 and Table 3, Figure 5). The atomic composition of the illite surface reveals a relatively low carbon content (Table 2), indicating that illite contains only a small amount of organic residue. Besides oxygen, silicon, and aluminum, XPS analysis detects the presence of potassium, sodium, and magnesium (Table 2).

Furthermore, the Si 2p region of illite reveals two distinct components at 102.4 eV and 103.7 eV (Figure 5b). These peaks correspond to Si 2p photoelectrons originating from Si^4+^ ions embedded in the tetrahedral SiO_4_^4−^ framework, and from surface Si^4+^ ions coordinated by OH^−^ groups [33]. Similarly, the Al 2p region exhibits two peaks at 74.4 eV and 76.3 eV (Figure 5c), which are attributed to Al^3+^ ions fixed within the octahedral aluminum oxide framework, and to surface Al^3+^ ions coordinated by OH^−^ groups [34]. In the K 2p region, two components are observed at 295.8 eV and 293.2 eV (Figure 5d), representing K 2p_1/2_ and 2p_3/2_ photoelectrons, respectively. This indicates a higher presence of free K^+^ ions compared to those that are fixed in the oxide structure [35].

Moreover, the C 1s region of illite exhibits three peaks at 284.7 eV, 286.0 eV, and 288.7 eV (Figure 5e), which correspond to saturated carbon (sp^3^ hybridization), carbon associated with C–O bonds, and carbon from carboxyl groups [36].

**Table 3:** The elemental and component compositions of illite and the crystalline sizes of illite’s components.

Elemental Mass Percentage (wt.%) ^a^				*d*_XRD_ (nm) (Component Mass Percentage, wt.%) ^b^		
K	Na	Mg	Al	KAl_2_Si_3_AlO_10_(OH)_2_	Na_2_MgSiO_4_	Al_2_SiO_4_(OH)_2_
8.30	0.10	0.06	10.18	36 nm (84.55 wt.%)	34 nm (0.40 wt.%)	20 nm (15.05 wt.%)

^a^ Determined by ICP–OES. ^b^ Crystalline sizes of illite components were determined by XRD (Figure 5f) according to Scherrer’s equation [37] using 0, 6, 0 (h, k, l) diffraction for KAl_2_Si_3_AlO_10_(OH)_2_ (illite), using 1, 1, 1 diffraction for Na_2_MgSiO_4_ (sodium magnesium silicate), and using 0, 2, 3 diffraction for Al_2_SiO_4_(OH)_2_ (aluminum silicate hydroxide). Component mass percentages were determined by ICP–OES.

Illite is composed of three crystalline phases as determined by XRD, as illustrated in Figure 5f. The first phase is illite-2M1, a sub-type of illite, with the chemical formula KAl_2_Si_3_AlO_10_(OH)_2_ (dark cubes in Figure 5f, PDF No. 26-0911). The second one is sodium magnesium silicate (Na_2_Mg_2_SiO_4_, blue cubes in Figure 5f, PDF No. 19-1216), while the final one corresponds to aluminate silicate hydroxide (Al_2_SiO_4_(OH)_2_, green cubes in Figure 5f, PDF No. 44-0269). Therefore, it appears that K^+^, Na^+^, and Mg^2+^ ions are dissociated from the [AlO_6_]^9−^ or [SiO_4_]^4−^ frameworks after ball milling.

**Figure 6:**
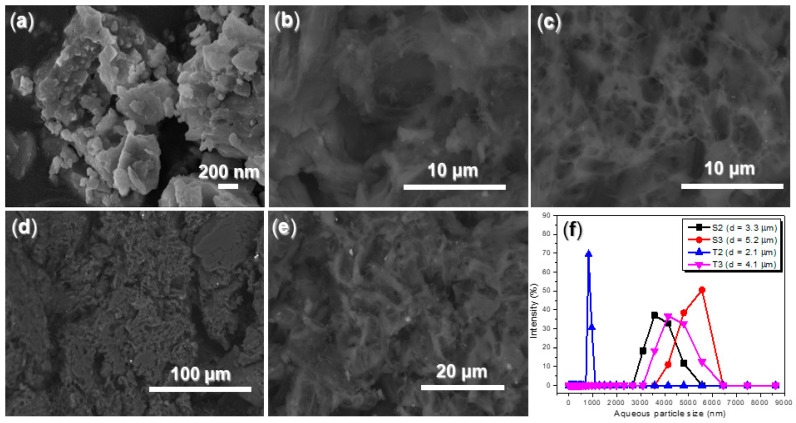
SEM images: (**a**) illite (magnification of 40,000×), (**b**) S2 (5000×), (**c**) T2 (5000×), (**d**) S3 (500×), (**e**) T3 (2000×). Aqueous particle sizes of admixtures: (**f**) S2 (black cubes), S3 (red dots), T2 (blue triangles), T3 (pink inverted triangles).

It is essential to quantify the phases of illite. As illustrated in Table 3, the KAl_2_Si_3_AlO_10_(OH)_2_ phase is the predominant component in illite, accounting for a mass percentage of 84.55% and exhibiting a particle size of 36 nm. The second largest component, Al_2_SiO_4_(OH)_2_, constitutes 15.05% by mass and has a particle size of 20 nm. Additionally, the Na_2_MgSiO_4_ phase has a mass percentage of 0.40% and a particle size of 34 nm. SEM reveals that the ball-milled illite consists of layered particles with sizes ranging from 10 to 400 nm (Figure 6a), encompassing the aforementioned three phases.

S2, the complex derived from the synthesized copolymer (S1, *p*(AA–*co*–MA–*co*–AMPS)) and AS, exhibits a fluffy, flocculent morphology (Figure 6b). When mixed with illite, the resulting admixture, S3, displays a wrinkled surface that encapsulates larger aggregates (Figure 6d). T2 presents a slightly denser morphology than S2, with smaller pores than those observed in S2 (Figure 6c vs. Figure 6b). Furthermore, the coating materials of T3 appear thinner than those of S3 (Figure 6e vs. Figure 6d), which reflects the structural differences between S1 and S2 (Figure 2).

S2 exhibits a smaller aqueous particle size compared to S3 (Figure 6f), indicating that illite remains hydrophobic, which facilitates the agglomeration of illite powders in water. T2 is smaller than S2 (Figure 6f), which can be attributed to their structural differences (Figure 2). Specifically, the prolonged carboxyl group linked by a methylene group in T2 promotes its agglomeration in water, resulting in the smaller micelles. Additionally, T3 is smaller than S3, likely for the same reason (Figure 6f).

### 3.2. Effects of Admixtures on Setting Times and Mechanical Strength Developments

The ISTs and FSTs of the cement pastes are summarized in Table 4, while the compressive and flexural strengths of the mortars are presented in Table 5. In the admixture-blank experiment, the cement coagulates very slowly (P1, Table 4). The introduction of AS as an admixture significantly reduces both the IST and FST (P2 vs. P1, Table 4). The presence of AS enhances both the compressive and flexural strengths of mortar at all time points compared to the admixture-blank trial (M2 vs. M1, Table 5). Obviously, the addition of AS provides more Al^3+^ ions, facilitating formation of [Al(OH)_4_]^−^, which may contribute to the proliferation of C_3_A, finally accelerating cementing [20].

The use of pure illite powder as an admixture significantly reduces both IST and FST compared to the admixture-blank experiment (P3 vs. P1, Table 4). However, it results in a decrease in the compressive strength of the mortar and shows no positive effect on flexural strength (M3 vs. M1, Table 5). This can be attributed to the chemical structure of illite (Figure 1c), which may release some dissociative Al^3+^ ions after ball milling [10,17], subsequently accelerating cement setting [20]. However, due to the hydrophobic nature of illite, the particles fail to penetrate the cement network effectively, hindering the development of mechanical strength.

The introduction of S1 results in a reduction in both IST and FST compared to the admixture-blank experiment (P4 vs. P1, Table 4). While the compressive strengths show improvement, flexural strengths decline (M4 vs. M1, Table 5). This outcome suggests that S1 acts as an acidic copolymer, effectively preventing the rapid hydrolysis of Al^3+^ into ineffective Al(OH)_3_ or Al_2_O_3_, which accelerates cementing and strengthens the cement network, thereby enhancing compressive strength [24]. However, the incorporation of S1 into the setting cement does not adequately fill the gaps or pores formed during cementing, resulting in the reduced flexural strength of the mortar.

In contrast, the most promising results arise from the S2-facilitated cement setting, which yields an IST of 2.44 min and an FST of 3.14 min (P5, Table 4). Additionally, significantly higher compressive and flexural strengths of the mortar are observed at all time points compared to those achieved with AS-facilitated mortar (M5 vs. M2, Table 5). Considering the composition of S2 (Figure 2), the coordinated Al^3+^ ions likely prevent ineffective hydrolysis into unreactive Al(OH)_3_ or Al_2_O_3_, thereby accelerating cement hydration and enhancing the compressive strength [24]. Furthermore, the ligand (S1) is highly hydrophilic, facilitating the encapsulation of AS and allowing it to fill the gaps or pores formed during the cementing process, which ultimately leads to improved flexural strength [22].

Reducing the dosage of admixture will extend the setting times (P6 vs. P5, Table 4), and both compressive and flexural strengths show a decline (M6 vs. M5, Table 5). Conversely, increasing the admixture dosage from 7 wt.% to 8 wt.% decreases both IST and FST (P7 vs. P5, Table 4), although this also results in a reduction in both compressive and flexural strengths (M7 vs. M5, Table 5). Herein, it is proposed that too much loading of AS will cause large formations of AFt on the surface of C_3_S, affecting the sufficient hydration of C_3_S into C-S-H [38,39]. This property may accelerate cementing, but meanwhile decrease the mechanical strength of the hydrated mortar. Therefore, the optimal admixture dosage is 7 wt.% relative to the cement content.

When the illite-based admixture (S3) is introduced, both IST and FST increase compared to those of S2 at the same dosage (P8 vs. P5, Table 4). However, both compressive and flexural strengths show an increase (M8 vs. M5, Table 5). Herein, the incorporation of ball-milled illite powders into S2 probably adsorbs Al^3+^ ions of AS more tightly, retarding cementing to some extent [20]. However, illite powders are induced to the pores and clefts of the cementing matrix, increasing the mechanical strengths of the mortar [22]. Furthermore, increasing the dosage of S3 reduces the IST (P9 vs. P8, Table 4), while both mechanical strengths decline (M9 vs. M8, Table 5). Clearly, an excessive dosage of illite-based admixture is harmful to cementing, probably because too much illite powder will inhibit the hydration of the C_3_S [38,39].

On the other hand, T2 exhibits faster cement setting times compared to S2 (P10 vs. P5, Table 4), and the compressive strengths at all time points are enhanced, but the flexural strengths remain nearly unchanged or decline (M10 vs. M5, Table 5). This difference can be attributed to the structural variations between T2 and S2 (Figure 2).

When maleic acid (MA) is replaced with itaconic acid (IA) as the copolymerization monomer, the extended carboxyl group linked by a methylene group may demonstrate improved plasticity during the cementing process, as previously observed [25]. This structural variation leads to better setting times and higher compressive strengths. However, it is likely that this change has a marginal or even negative impact on the hydrophilicity of the copolymer, which does not enhance the flexural strength of the mortar.

Another possible reason is that the aqueous particle size of T2 is significantly smaller than that of S2 (Figure 6f). This reduced particle size may facilitate the cementing process due to the increased surface area, resulting in a faster release of Al^3+^ ions. Additionally, the improved compressive strength can be attributed to the quicker hydration of C_3_S into C-S-H, which is accelerated by the smaller particle size. However, smaller particles may not effectively fill the pores and gaps within the cementing matrix compared to larger particles, leading to a lower flexural strength of the mortar.

Increasing the dosage of T2 has minimal effects on setting times (P11 vs. P10, Table 4) and negatively impacts the development of compressive strength. However, it does improve flexural strength at all time points (M11 vs. M10, Table 5). Herein, after enough coated Al^3+^ ions for rapid cementing are provided, increasing the excessive dosage of admixture mainly means filling more gaps and pores formed during cementing processes.

When T3 is used as an admixture, both setting times are prolonged compared to the use of T2 (P12 vs. P10, Table 4), and compressive strengths are reduced too. However, the flexural strengths of the mortar at all time points show a significant increase (M12 vs. M10, Table 5). This observation further clarifies that the coated illite primarily acts as an adhesive filling agent in the cement setting, influencing flexural strength more than contributing to the formation of the fundamental cement skeleton [22].

Both increasing and decreasing the dosage of T3 lead to longer setting times for the pastes (P13–14 vs. P12, Table 4). The compressive strengths of the mortar with a higher dosage of 8 wt.% T3 are lower than those with a lower dosage of 7 wt.% (M13 vs. M12, Table 5). However, notably, the use of T3 at the 8 wt.% dosage results in increased flexural strengths at all time points compared to the 7 wt.% dosage (M13 vs. M12, Table 5). This observation reinforces the idea that coated illite primarily functions as an adhesive filling agent in the cement setting process [22].

**Figure 7:**
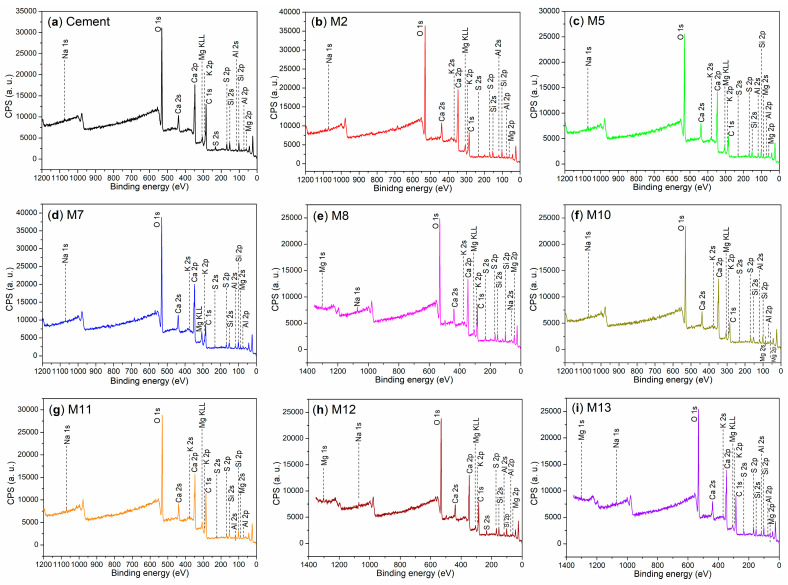
XPS survey scans: (**a**) cement, (**b**) M2, (**c**) M5, (**d**) M7, (**e**) M8, (**f**) M10, (**g**) M11, (**h**) M12, (**i**) M13; all mortars were tested at 24 h.

**Figure 8:**
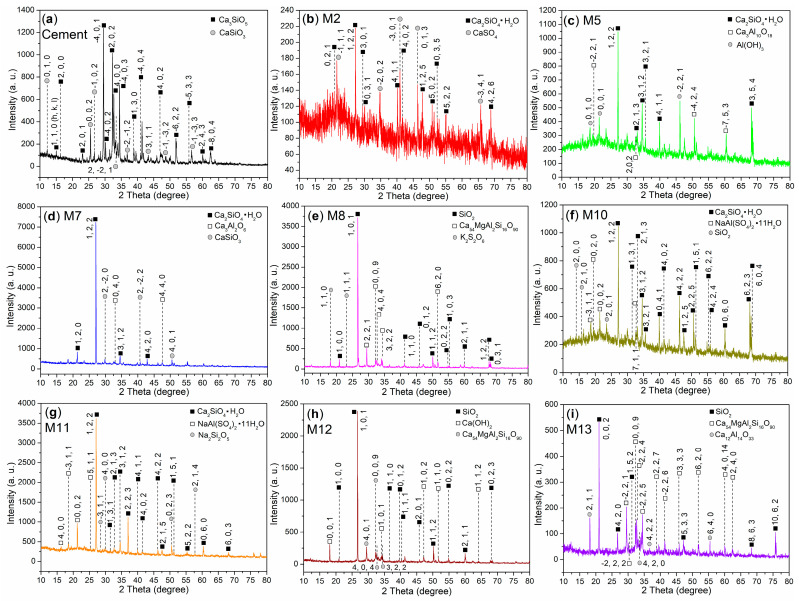
Wide-angle (2*θ* = 10–80°) XRD spectra: (**a**) cement, (**b**) M2, (**c**) M5, (**d**) M7, (**e**) M8, (**f**) M10, (**g**) M11, (**h**) M12, (**i**) M13; all mortars were tested at 24 h.

On the other hand, T3 outperforms S3 in both setting times (P12 vs. P8, Table 4) and mechanical strengths (M12 vs. M8, Table 5). This enhanced performance can be attributed to T3’s smaller particle size compared to S3 (Figure 6f), which is influenced by the differing structures of T1 and S1. Specifically, T1 appears to be more lipophilic than S1 due to the presence of a methylene spacer in the IA monomer (Figure 2). Additionally, the evolution of T3 during the cementing process of mortar likely differs from that of S3 (Figure 7e,h and Figure 8e,h), resulting in variations in the mechanical strengths of the mortar.

Additionally, M8 exhibits a higher compressive strength retention ratio (*R*_28_) than M5, and M12 also appears to outperform M10 (Table 6). This result suggests that the incorporation of coated illite contributes to the long-term mechanical strength development of mortar when compared to the use of aluminum-containing admixtures.

According to the Chinese standard GB/T 35159-2017 [40], the requirements stipulate that the IST should be ≤5 min, FST should be ≤12 min, the compressive strength of mortar at 24 h must exceed 7.0 MPa, and the retention ratio at 28 days (*R*_28_) should be greater than 90% to ensure the effective use of admixtures in shotcrete. Based on these criteria, both T2 and T3 appear to meet the necessary standards (P10, P12, Table 4; M10, M12, Table 5).

### 3.3. Microstructural Characterizations of Cement and Mortars

To gain a deeper understanding of the cement hydration processes influenced by various admixtures, both cement and mortars are thoroughly characterized. The FT-IR spectrum of cement is presented in Figure 4h. Firstly, the moderate peak observed at 3716 cm^−1^ can be attributed to the O–H vibration of hydroxyl groups on the surface of metal oxides in the cement. The subsequent peak at 3048 cm^−1^ indicates C–H aromatic stretching bands, which likely originate from organic residues present in the cement. Additionally, the two small peaks at 2940 cm^−1^ and 2837 cm^−1^ correspond to the anti-symmetric and symmetric stretching vibrations of methylene groups [30], also representing the organic residues.

Secondly, the peak observed at 1446 cm^−1^ is characteristic of C–C stretching vibrations associated with organic residues [30]. The cement further displays two peaks at 1144 cm^−1^ and 955 cm^−1^, which correspond to the anti-symmetric and symmetric stretching vibrations of SO_4_^2−^ from CaSO_4_ present in the cement [41]. Additionally, a small peak at 682 cm^−1^ corresponds to the in-plane bending vibrations of C–O bonds [30]. Lastly, the peaks observed at 602 cm^−1^, 522 cm^−1^, and 447 cm^−1^ can be attributed to the stretching vibrations of Mg–O, Ca–O, and Al–O bonds, respectively.

The bulk chemical composition of cement is presented in Table 7, while the binding energies and atomic compositions on the surfaces of the cement and mortars are detailed in Table 8. The XPS survey scans of these materials are illustrated in Figure 7. XRD analysis reveals that the cement is composed of two phases (Figure 8a). The first phase is calcium silicate (Ca_3_SiO_5_, 3CaO·SiO_2_, C_3_S, represented by dark cubes, PDF No. 49-0442, Figure 8a), and the second is wollastonite-1A (CaSiO_3_, CaO·SiO_2_, CS, indicated by gray circles, PDF No. 29-0372, Figure 8a). This composition is consistent with that of typical OPC [42].

When AS is used as an admixture, the resulting mortar (M2) exhibits two distinct phases. The first phase is calcium silicate hydrate (Ca_2_SiO_4_·H_2_O, 2CaO·SiO_2_·H_2_O, C_2_SH, dark cubes, PDF No. 29-0373, Figure 8b), which appears to be a precursor to the final hydrated product, C–S–H (3CaO·2SiO_2_·3H_2_O). The second phase is calcium sulfate (CaSO_4_, gray circles, PDF No. 30-0279, Figure 8b), originating from the cement and not yet transformed into AFt (calcium sulfoaluminate hydrate, 3CaO·Al_2_O_3_·3CaSO_4_·32H_2_O). This lack of transformation may be attributed to the insufficient reactivity of pure AS to accelerate the cementing effectively.

However, when AS is replaced with S2 as an admixture, the composition of the resulting M5 undergoes significant changes compared to M2. Although M5 still exhibits the C_2_SH phase present in M2 (Figure 8c vs. Figure 8b), the CaSO_4_ phase is absent, and two new phases emerge. The first one is calcium aluminum oxide (Ca_3_Al_10_O_18_, 3CaO·5Al_2_O_3_, hollow cubes, PDF No. 01-0572, Figure 8c), probably a derivative of C_3_A (calcium aluminum oxide, Ca_3_Al_2_O_6_, 3CaO·Al_2_O_3_), formed by the addition of four more Al_2_O_3_ units. The second is doyleite (Al(OH)_3_, gray circles, PDF No. 38-0376, Figure 8c), likely resulting from the hydrolysis of the Al^3+^ ions of S2 (Figure 2).

Consequently, the reduced setting times of P5 compared to P2 (Table 4) and the improved compressive and flexural strengths of M5 relative to M2 (Table 5) can be attributed to the adhesive properties of these two newly identified phases, namely 3CaO·5Al_2_O_3_ and Al(OH)_3_. Notably, the adhesive properties of 3CaO·5Al_2_O_3_, a new clinker, are reported for the first time in this work.

When the dosage of S2 is increased from 7 wt.% to 8 wt.%, the composition of the resulting M7 changes once again. In addition to the C_2_SH phase, M7 displays C_3_A (Ca_3_Al_2_O_6_, 3CaO·Al_2_O_3_, hollow cubes, PDF No. 32-0148, Figure 8d), as well as wollastonite-1A (CaSiO_3_, CaO·SiO_2_, CS, gray circles, PDF No. 29-0372, Figure 8d). The former phase is commonly observed in cementitious materials and often acts as a clinker to reinforce the C–S–H network [44]. The latter phase is likely residual unreacted CS from the cement (Figure 8d vs. Figure 8a).

It is noteworthy that the dosage of S2 at 8 wt.% results in reduced setting times compared to 7 wt.% (P7 vs. P5, Table 4), as well as decreased mechanical strengths (M7 vs. M5, Table 5). This indicates that the excessive loading of S2 as an admixture promotes the formation of C_3_A while hindering the transformation of CS, ultimately inhibiting the cementing process.

When the illite-based admixture (S3) is introduced, the composition of the resulting M8 undergoes unexpected changes. Initially, the C_2_SH phases, which are prevalent in M2, M5, and M7, disappear, and instead, quartz (low) becomes the dominant aggregate (SiO_2_, dark cubes, PDF No. 65-0466, Figure 8e) in M8. It is likely that the C_2_SH has been completely transformed into C–S–H gel, which exhibits no significant diffractions in the range of 2*θ* = 10–80° [45].

Furthermore, a new phase, calcium magnesium aluminum oxide silicate (Ca_54_MgAl_2_Si_16_O_90_, 54CaO·MgO·Al_2_O_3_·16SiO_2_, hollow cubes, PDF No. 13-0272, Figure 8e), emerges, with its MgO component potentially derived from the coated illite-based admixture (S3, Figure 2).

Although the incorporation of S3 results in prolonged setting times compared to S2 (P8 vs. P5, Table 4), both the compressive and flexural strengths improve (M8 vs. M5, Table 5). This enhancement is likely due to the formation of the Ca_54_MgAl_2_Si_16_O_90_ phase, as well as the complete hydration of the C_2_SH phase into C–S–H gel.

Previous studies have reported that the addition of highland barley straw ash (HBSA) into magnesium oxychloride cement (MOC) leads to the formation of magnesium silicate hydrate gel, which enhances flexural and compressive strengths, as well as physical and working performance, by modifying the pore structure and improving compactness [46]. Clearly, the new clinker phase, Ca_54_MgAl_2_Si_16_O_90_, identified in this study exhibits similar beneficial cementing effects.

Additionally, another new phase identified is potassium sulfate (K_2_S_2_O_6_, gray circles, PDF No. 15-0236, Figure 8e), which likely originates from the illite-based admixture (as a K^+^ source) and sulfur-containing species present in the cement.

On the other hand, the introduction of T2 results in shorter setting times compared to S2 at the same dosage of 7 wt.% (P10 vs. P5, Table 4). This improvement may be attributed to the copolymer T2 (T1), which contains extended carboxyl groups linked by methylene groups, potentially providing better coordination for stabilizing Al^3+^ ions than the carboxyl groups found in the copolymer S2 (S1) (Figure 2).

Furthermore, while the compressive strengths at all time points show improvement, the effects on flexural strengths are negative (M10 vs. M5, Table 5). In this context, M10, the mortar enhanced by T2, exhibits the same dominant phase of C_2_SH as M5 (Figure 8f vs. Figure 8c). However, M10 also reveals two new phases not present in M5: mendozite (NaAl(SO_4_)_2_·11H_2_O, hollow cubes, PDF No. 22-0475, Figure 8f) and silicon oxide (SiO_2_, gray circles, PDF No. 32-0993, Figure 8f). It appears that these two in situ-formed phases may contribute to the setting times (e.g., Al^3+^ release from mendozite) and the development of compressive strength (e.g., the SiO_2_ clinker). However, they may not effectively fill the gaps or pores in the mortar during the cementing process.

Moreover, increasing the dosage of T2 from 7 wt.% to 8 wt.% results in only marginal effects on setting times (P11 vs. P10, Table 4) while causing a decrease in compressive strengths. However, it enhances flexural strengths at all time points (M11 vs. M10, Table 5). The only compositional difference between M11 and M10 is that M11 contains the natrosilite phase (Na_2_Si_2_O_5_, gray circles in Figure 8g), whereas M10 contains the SiO_2_ phase (Figure 8f). Natrosilite appears to be softer and more pliable than SiO_2_, which may contribute to the improved flexural strength observed in M11, while simultaneously leading to a reduction in compressive strength.

It is also intriguing to examine the effects of the admixture (T3) derived from the combination of T2 and illite. Initially, M12, the mortar enhanced by T3, exhibits a composition very similar to that of M8, which is enhanced by S3 (Figure 8e,h). The sole distinction between M12 and M8 is that M12 contains portlandite (Ca(OH)_2_, CH, hollow cubes, PDF No. 04-0733, Figure 8h), while M8 contains K_2_S_2_O_6_ (Figure 8e). Furthermore, portlandite is a typical hydrated product in cementitious materials; its presence indicates a higher degree of cement hydration [47], which in turn results in faster setting times and greater compressive and flexural strengths (M12 vs. M8, Table 5).

Moreover, increasing the dosage of T3 from 7 wt.% to 8 wt.% leads to the formation of mayenite (Ca_12_Al_14_O_33_, 12CaO·7Al_2_O_3_, gray circles in Figure 8i) in M13, as opposed to the greater presence of portlandite in M12 (Figure 8e). Concurrently, the increased dosage of T3 results in prolonged setting times (P13 vs. P12, Table 4) and reduced compressive strengths, while flexural strengths improve at all time points (M13 vs. M12, Table 5). Clearly, the amount of coated illite used as an admixture is highly sensitive to the effects of cement hydration; as the quantity of coated illite increases, a higher flexural strength is achieved.

### 3.4. Analysis of Chemical States of Elements in Mortars

**Figure 9:**
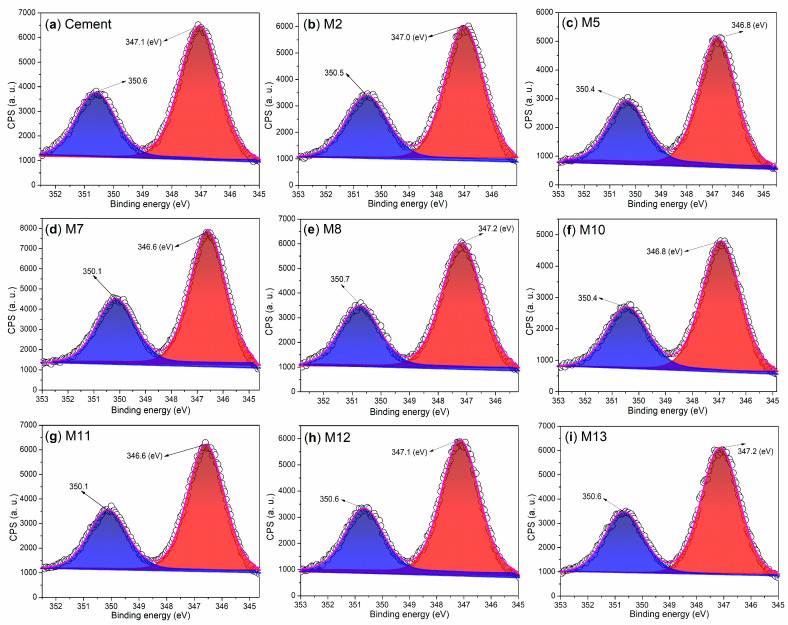
XPS measurements of Ca 2p regions: (**a**) cement, (**b**) M2, (**c**) M5, (**d**) M7, (**e**) M8, (**f**) M10, (**g**) M11, (**h**) M12, (**i**) M13; all mortars were tested at 24 h.

To further confirm the chemical compositions of the mortars identified by XRD, we conducted an investigation into the chemical states of the elements of the mortars using XPS. Initially, we examined the Ca 2p regions of both the cement and mortars, as depicted in Figure 9. The cement exhibits two distinct peaks at 350.6 eV and 347.1 eV (Figure 9a), which correspond to the Ca 2p_1/2_ and Ca 2p_3/2_ photoelectrons, respectively [48]. These peaks are indicative of the Ca^2+^ ions associated with non-hydrated phases of C_3_S and CS (Figure 8a).

When non-illite-type admixtures, such as AS, S2, and T2, are incorporated into the cement, the resulting mortars—M2, M5, M7, M10, and M11—exhibit lower binding energies for Ca 2p_1/2_ and Ca 2p_3/2_ compared to those observed in cement (Figure 9b–d,f,g vs. Figure 9a). This observation primarily indicates that the C_3_S and CS components in cement have undergone hydration, transforming into C_2_SH. This transformation has been confirmed by XRD analysis in M2 (Figure 8b), M5 (Figure 8c), M7 (Figure 8d), M10 (Figure 8f), and M11 (Figure 8g).

In contrast, when illite-based admixtures such as S3 and T3 are introduced, the resulting mortars—M8, M12, and M13—exhibit equal or higher binding energies for Ca 2p_1/2_ and Ca 2p_3/2_ compared to those observed in cement (Figure 9e,h,i vs. Figure 9a). Correlating with the XRD results, it is evident that the formation of Ca_54_MgAl_2_Si_16_O_90_ (54CaO·MgO·Al_2_O_3_·16SiO_2_) in M8 (Figure 8e), M12 (Figure 8h), and M13 (Figure 8i), as well as Ca(OH)_2_ in M12 (Figure 8h) and mayenite (Ca_12_Al_14_O_33_, 12CaO·7Al_2_O_3_) in M13 (Figure 8i), contributes to the observed changes in the binding energies of the Ca 2p_1/2_ and Ca 2p_3/2_ photoelectrons.

**Figure 10:**
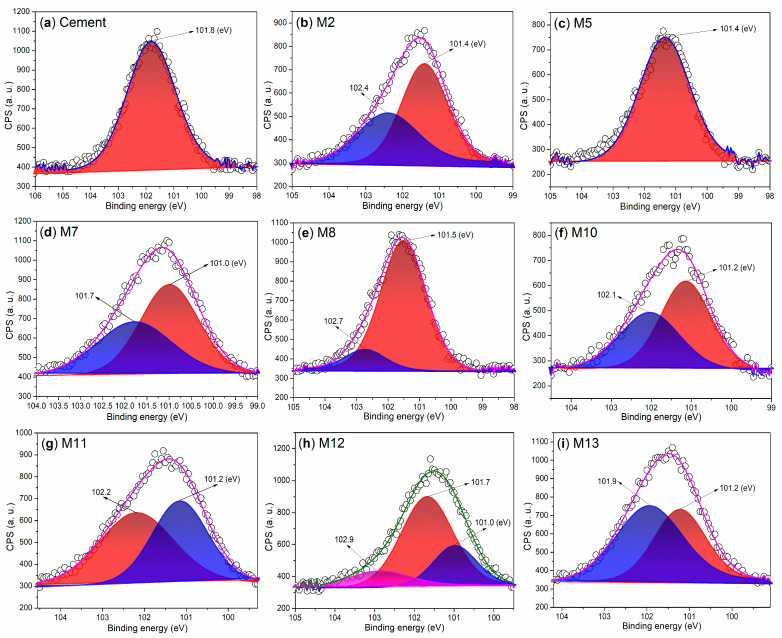
XPS measurements of Si 2p regions: (**a**) cement, (**b**) M2, (**c**) M5, (**d**) M7, (**e**) M8, (**f**) M10, (**g**) M11, (**h**) M12, (**i**) M13; all mortars were tested at 24 h.

It is also noteworthy to examine the chemical states of silicon in the hydrated mortars. The cement displays a Si 2p peak at 101.8 eV (Figure 10a), which corresponds to the silicon of C_3_S and CS (Figure 8a) [33]. M2 exhibits two Si 2p peaks at 101.4 eV and 102.4 eV (Figure 10b), indicating the presence of silicon in C_2_SH and amorphous SiO_2_, respectively.

In contrast, only a single peak appears at 101.4 eV in the Si 2p region of M5 (Figure 10c), which characterizes the silicon in C_2_SH. This finding further suggests that the use of S2 as an admixture significantly accelerates the hydration of SiO_2_ compared to that observed with pure AS (Figure 10c vs. Figure 10b).

M7 displays two Si 2p peaks at 101.0 eV and 101.7 eV (Figure 10d). The first peak, at 101.0 eV, is characteristic of the silicon in C_2_SH, while the second peak at 101.7 eV corresponds to the silicon in CS (Figure 8d). M8 also exhibits two Si 2p peaks at 101.5 eV and 102.7 eV (Figure 10e), which are associated with the silicon in SiO_2_ and the silicon in Ca_54_MgAl_2_Si_16_O_90_, respectively (Figure 8e).

When T2 is used as an admixture, the resulting M10 exhibits two peaks in the Si 2p region at 101.2 eV and 102.1 eV (Figure 10f). The first peak at 101.2 eV is indicative of the silicon in C_2_SH, while the second peak at 102.1 eV corresponds to the silicon in SiO_2_ (Figure 8f). When the loading amount of T2 is increased, the resulting M11 displays a similar spectral profile to that of M10; however, the peak at 102.2 eV is now associated with the silicon in Na_2_Si_2_O_5_ rather than SiO_2_ (Figure 10g vs. Figure 10f, Figure 8g vs. Figure 8f).

When an illite-based admixture (S3) is incorporated, the resulting M12 exhibits peaks at 101.0 eV, 101.7 eV, and 102.9 eV (Figure 10h). These peaks correspond to the silicon from amorphous SiO_2_, crystalline SiO_2_, and Ca_54_MgAl_2_Si_16_O_90_, respectively (Figure 8h). As the dosage of S3 is increased, two additional peaks appear at 101.2 eV and 101.9 eV in the Si 2p region of the resulting M13 (Figure 10i). These peaks characterize the silicon from SiO_2_ and Ca_54_MgAl_2_Si_16_O_90_ (Figure 8i).

**Figure 11:**
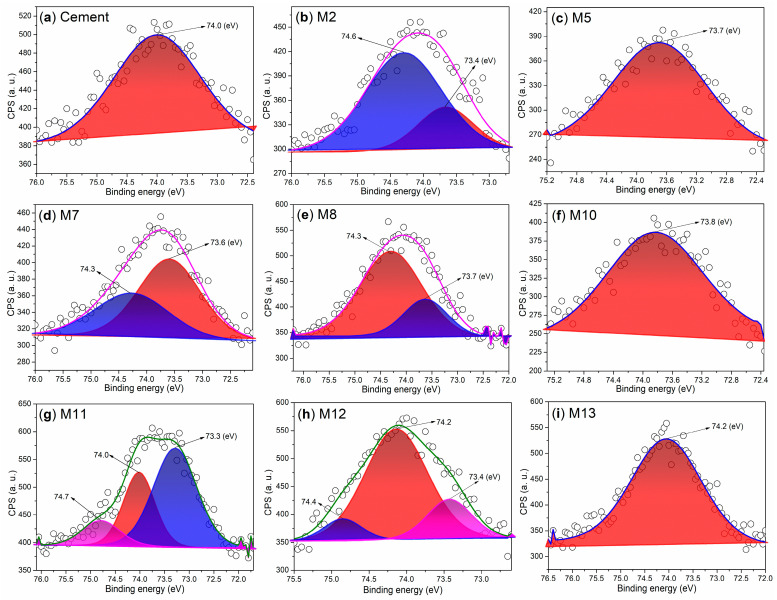
XPS measurements of Al 2p regions: (**a**) cement, (**b**) M2, (**c**) M5, (**d**) M7, (**e**) M8, (**f**) M10, (**g**) M11, (**h**) M12, (**i**) M13; all mortars were tested at 24 h.

The chemical states of aluminum in mortars also warrant attention. Initially, the cement exhibits a single Al 2p peak at 74.0 eV (Figure 11a), primarily indicating the presence of Al_2_O_3_ [34]. After 24 h of hydration facilitated by AS, two peaks emerge at 73.4 eV and 74.6 eV (Figure 11b), likely corresponding to various hydrated aluminum oxides. However, when S2 is used as an admixture, the Al 2p peak becomes more pronounced at 73.7 eV (Figure 11c), predominantly associated with Ca_3_Al_10_O_18_ (Figure 8c).

As the dosage of S2 is increased, the resulting M7 exhibits two Al 2p peaks at 73.6 eV and 74.3 eV (Figure 11d), which correspond to C_3_A and other aluminum-containing compounds with low crystallinity (Figure 8d). When an illite-based admixture (S3) is introduced, two peaks appear at 73.7 eV and 74.3 eV (Figure 11e), indicating the presence of aluminum from amorphous hydrated products and from Ca_54_MgAl_2_Si_16_O_90_ (Figure 8e).

When T2 is used as an admixture, the Al 2p signal is concentrated in a single peak at 73.8 eV (Figure 11f), indicating the presence of aluminum from NaAl(SO_4_)_2_·11H_2_O in M10 (Figure 8f). As the dosage of T2 is increased, the resulting M11 displays multiple peaks in the Al 2p region (Figure 11g). In addition to the peak at 73.3 eV, which characterizes NaAl(SO_4_)_2_·11H_2_O, there are also two additional peaks representing other aluminum-containing components.

**Figure 12:**
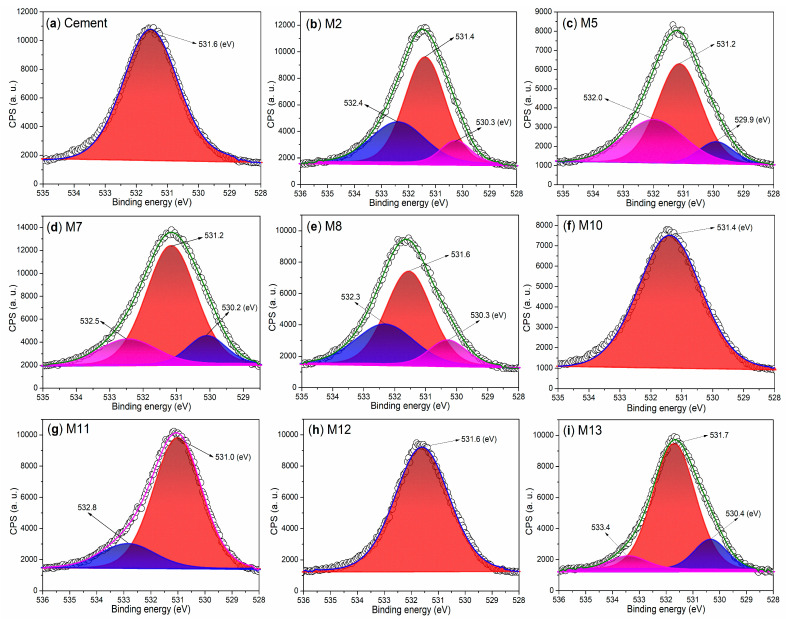
XPS measurements of O 1s regions: (**a**) cement, (**b**) M2, (**c**) M5, (**d**) M7, (**e**) M8, (**f**) M10, (**g**) M11, (**h**) M12, (**i**) M13; all mortars were tested at 24 h.

If another illite-based admixture (T3) is used, in addition to the peak which appears at 74.2 eV, indicating aluminum of Ca_54_MgAl_2_Si_16_O_90_ (Figure 8h), the other two peaks indicate other aluminum-containing components (Figure 11h). When the loading amount of T3 is increased, the resulting M13 just displays one peak centered at 74.2 eV (Figure 12i), covering the effects from not only Ca_54_MgAl_2_Si_16_O_90_, but also Ca_12_Al_14_O_33_ (Figure 8i).

From the perspective of anions, the cement exhibits only one O 1s peak (Figure 12a), reflecting the contributions of oxygen from C_3_S and CS (Figure 8a). The O 1s region of M2 displays three peaks at 532.4 eV, 531.4 eV, and 530.3 eV (Figure 12b), corresponding to the oxygen from crystallized H_2_O, SiO_2_, and CaO, respectively (Figure 8b) [49]. When S2 and S3 are used as admixtures, the resulting mortars, including M5, M7, and M8, exhibit similar profiles to M2 (Figure 12c–e vs. Figure 12b), illustrating the distribution of oxygen among various hydrated mortars (Figure 8c–e vs. Figure 8b).

In the case of the T2-facilitated hydrated mortars (M10 and M11), the single peak observed at 531.4 eV in the O 1s region of M10 encompasses the contributions of oxygen from C_2_SH, sulfate, and SiO_2_ (Figure 8f and Figure 12f). M11 displays an additional peak at 532.8 eV (Figure 12g), which can be attributed to the oxygen of hydroxyl groups on the copolymers (Figure 2).

When the illite-based admixture (T3) is utilized, the resulting mortar (M12) displays a single band centered at 531.6 eV (Figure 12h), indicating the contributions of various oxygen-containing components (Figure 8h). As the dosage of T3 is increased, three peaks emerge at 533.4 eV, 531.7 eV, and 530.4 eV (Figure 12i), corresponding to the oxygen from organic components, SiO_2_, and metal oxide, respectively (Figure 8i).

### 3.5. Morphological Characterizations of Mortars

**Figure 13:**
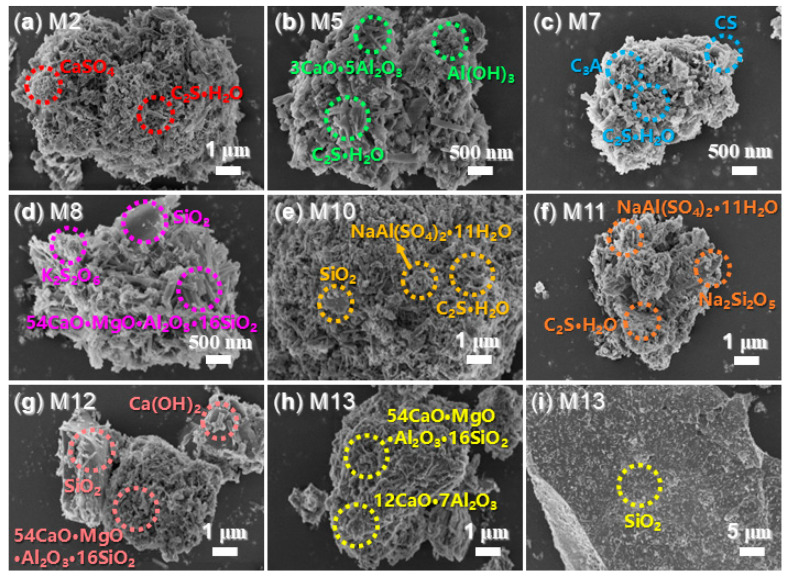
SEM images of mortars (24 h): (**a**) M2 (magnification of 10,000×), (**b**) M5 (20,000×), (**c**) M7 (20,000×), (**d**) M8 (20,000×), (**e**) M10 (10,000×), (**f**) M11 (10,000×), (**g**) M12 (10,000×), (**h**) M13 (10,000×), (**i**) M13 (2000×); all mortars were tested at 24 h.

Testing the morphologies of hydrated mortars is both significant and intriguing. Firstly, as illustrated in Figure 13a, M2 is composed of small particles ranging from 50 to 80 nm, corresponding to CaSO_4_ [50], as well as fibers measuring 0.5 to 1 μm in length and 80 to 100 nm in diameter, which can be attributed to C_2_SH (C_2_S·H_2_O, Figure 8b) [51]. Additionally, M5 not only contains C_2_SH but also exhibits the phase of Ca_3_Al_10_O_18_ (3CaO·5Al_2_O_3_), characterized by isolated fibers with lengths of 300 to 500 nm (Figure 8c and Figure 13b). Furthermore, the much smaller particles observed in the SEM of M5 can be ascribed to Al(OH)_3_ (Figure 8c and Figure 13b).

M7 not only exhibits the phase of C_2_SH but also displays C_3_A, which has a fiber-like morphology smaller than that of Ca_3_Al_10_O_18_ (Figure 8d and Figure 13c) [52]. Additionally, the CS phase in M7 is characterized by a layered structure with a scale of 300 to 500 nm (Figure 13c), a morphological property that has been corroborated by previous findings [53].

In contrast, both M5 and M7 present a denser appearance compared to M2, with numerous gaps and pores filled by undesirable species in M5 and M7, unlike those observed in M2 (Figure 13b,c vs. Figure 13a). Consequently, the use of AS as an admixture (resulting in M2) leads to significantly longer setting times and much lower mechanical strengths compared to those obtained from the use of S2 (resulting in M5 and M7) (P2 vs. P5 and P7, Table 4; M2 vs. M5 and M7, Table 5).

M8 exhibits a markedly different morphology compared to M2, M5, and M7. The larger blocks with smooth surfaces can be attributed to SiO_2_, while the much smaller particles appear to be K_2_S_2_O_6_ (Figure 8e and Figure 13d). Additionally, the significantly larger and bolder fibers, measuring between 300 nm and 1 μm in length and approximately 100 nm in diameter, correspond to Ca_54_MgAl_2_Si_16_O_90_ (Figure 8e and Figure 13d).

It is evident that the in situ-formed SiO_2_ blocks are tightly bound to the Ca_54_MgAl_2_Si_16_O_90_ phase (Figure 10d), and this interaction is characterized by covalent bonding rather than weaker physical adsorption, as indicated by the higher binding energies of the Ca 2p and Si 2p photoelectrons in M8 compared to those in cement, M2, M5, and M7 (Figure 9 and Figure 10). Therefore, it is clear that Ca_54_MgAl_2_Si_16_O_90_ acts as an active clinker with excellent pozzolanic activity.

When T2 is used as an admixture, the resulting M10 exhibits significantly longer C_2_SH fibers compared to those in M5 (Figure 13e vs. Figure 13b). This difference may account for the higher compressive strengths observed in M10 compared to M5 (Table 5). Furthermore, increasing the amount of T2 contributes to the formation of Na_2_Si_2_O_5_ (Figure 8g and Figure 13f), which may lead to a decrease in compressive strength while simultaneously enhancing flexural strength (M11 vs. M10, Table 5).

When another illite-based admixture (T3) is used, the resulting M12 exhibits a morphology similar to that of M8, which utilizes S3 as an admixture (Figure 13g vs. Figure 13d). However, M12 contains Ca(OH)_2_ rather than the K_2_S_2_O_6_ found in M8 (Figure 13g vs. Figure 13d). It is well established that Ca(OH)_2_ acts as an active clinker with high pozzolanic activity, which explains why M12 demonstrates shortened setting times and significantly improved compressive and flexural strengths compared to M8 (P12 vs. P8, Table 4; M12 vs. M8, Table 5). Clearly, T3 exhibits greater reactivity than S3 in activating illite during the formation of the cement admixture.

As the dosage of T3 is increased, the resulting M13 contains Ca_54_MgAl_2_Si_16_O_90_, mayenite (Ca_12_Al_14_O_33_), as well as SiO_2_ (Figure 8i and Figure 13h,i). Previous studies have indicated that the calcined mayenite enhances cement hydration due to its affinity for gypsum. Therefore, in addition to Ca_54_MgAl_2_Si_16_O_90_, the in situ formation of mayenite contributes to the improved flexural strength of the hydrated mortar (M13 vs. M12, Table 5).

It is worth delving deeper into the sizes and proportions of SiO_2_ and Ca_54_MgAl_2_Si_16_O_90_ in the hydrated mortars. Firstly, all the hydrated mortars enhanced by illite-based admixtures exhibit SiO_2_ sizes ranging from 82 to 102 nm (Table 9), which serve as fundamental units for the construction of larger bulk SiO_2_ blocks observed in SEM (Figure 13d,g–i). Furthermore, the Ca_54_MgAl_2_Si_16_O_90_ phases display sizes between 42 and 57 nm (Table 9), also functioning as building blocks for the formation of larger structures detected by SEM (Figure 13d,g–i). Additionally, according to ICP-OES analysis, all hydrated mortars (M8, M12, M13) contain mass percentages of Ca_54_MgAl_2_Si_16_O_90_ ranging from 52 to 68 wt.% (Table 9), indicating that Ca_54_MgAl_2_Si_16_O_90_ is the predominant component in hydrated mortars facilitated by illite-based admixtures.

### 3.6. Thermal and Functional Group Characterizations of Mortars

**Figure 14:**
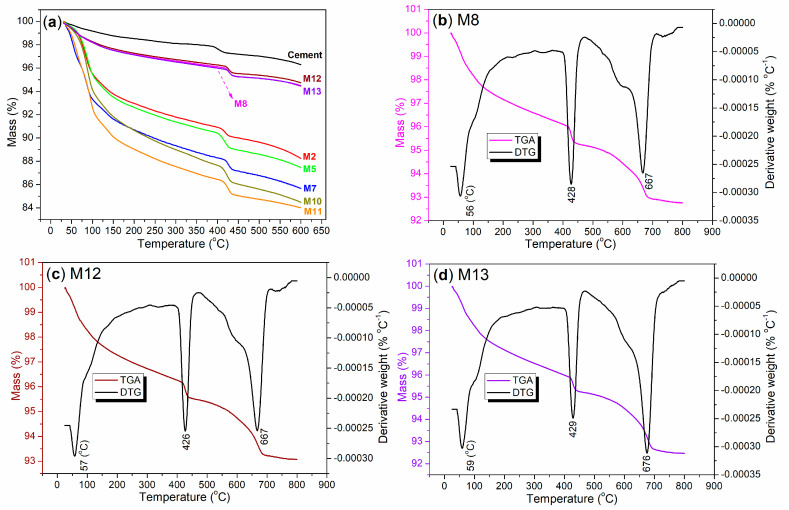
Thermal analyses of cement and mortars (hydrated for 24 h): (**a**) TGA curves of cement and mortars, (**b**) TGA and DTG curves of M8, (**c**) TGA and DTG curves of M12, (**d**) TGA and DTG curves of M13.

It is also intriguing to examine the thermal properties of hydrated mortars, as this analysis can provide insights into the presence of organic residues within mortars. First, TGA of cement and mortars was conducted at 30–600 °C (Figure 14a), and cement exhibited the lowest weight loss (black line, Figure 14a). The weight loss observed between 30 and 200 °C can be attributed to the release of adsorbed or coordinated water upon heating, while the weight loss occurring between 200 and 600 °C corresponds to the evaporation of organic residues as the temperature increases.

Next, at 30–600 °C, the total weight loss order of the mortars is as follows: M12 < M8 = M13 < M2 < M5 < M7 < M10 < M11 (Figure 14a). Based on this order, the mortars can be classified into four distinct groups. The first group includes M12, M8, and M13 (wine, magenta, violet lines, Figure 14a), all of which are mortars enhanced by illite-based admixtures (Table 5, Figure 2). It is evident that the incorporation of illite-based admixtures facilitates the filling of gaps or pores within the cement network formed during hydration. However, it is also important to note that when illite fills these gaps or pores, the hydrophilic organic components of the admixtures are excluded from the mortar.

The second group consists of M2 (red line, Figure 14a), which is a pure AS-facilitated mortar. This mortar contains more volatiles than those in the first group, but less than other mortars. In this context, AS refers to a water-soluble salt, and its dispersion into the gaps or pores of the hydrated mortar may occur in situ. This means that the Al^3+^ ions may react with other components present in the pores, resulting in the formation of fillings. Consequently, this process leads to an increase in crystallized water while simultaneously reducing the amount of solid fill material within the mortar.

Group 3 includes two mortars, M5 and M7 (green and blue lines in Figure 14a), both of which are enhanced with S2 as an admixture (Table 5). The dosage of S2 in M5 is lower than that in M7 (Table 5), which results in the TGA curve for M5 consistently remaining higher than that for M7 at 30–600 °C (Figure 14a). This observation indicates that a greater amount of admixture leads to an increased volume of filling materials residing within the pores of the cement.

The final group includes M10 and M11 (dark yellow and orange lines in Figure 14a). Initially, M10 and M11 exhibit greater total weight losses at 30–600 °C compared to M5 and M7, respectively. This suggests that T2 demonstrates superior filling effects during cement hydration when compared to S2 (Table 5, Figure 2). Additionally, the TGA curve for M10 consistently remains higher than that for M11, further indicating that a higher dosage of T2 results in an increased amount of filling materials.

To further investigate the structural differences among M8, M12, and M13, TGA and DTG curves were obtained with the heating temperature increased to 800 °C (Figure 14b–d). M8 exhibits three distinct weight loss rates at 56 °C, 428 °C, and 667 °C (Figure 14b), which closely resemble those observed in M12 (Figure 14c). This suggests that the volatile compositions of M8 and M12 are quite similar. In contrast, M13 displays weight loss rates at 59 °C, 429 °C, and 676 °C (Figure 14d), likely due to the formation of mayenite (Ca_12_Al_14_O_33_, Figure 8i).

**Figure 15:**
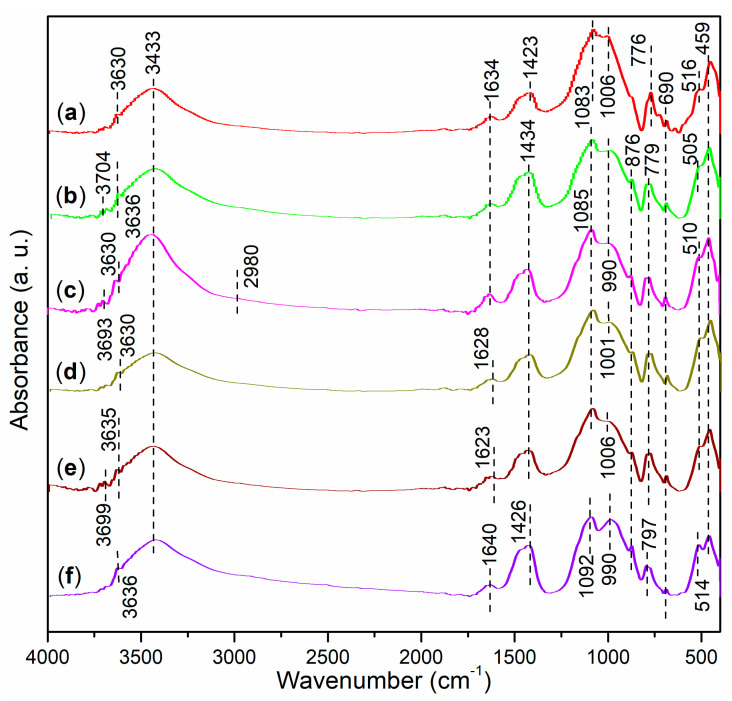
FT-IR spectra (adsorption mode) of mortars (hydrated for 24 h): (**a**) M2, (**b**) M5, (**c**) M8, (**d**) M10, (**e**) M12, (**f**) M13.

It is also intriguing to investigate the functional groups present in hydrated mortars using FT-IR spectroscopy, as this may provide insights into the organic constituents of the mortars. Notably, M2 exhibits a small peak at 3630 cm^−1^, which corresponds to the O–H stretching vibration of the hydroxyl groups located on the surface of metal oxides, such as the CaO unit in M2 (Figure 15a) [30]. The subsequent peak at 3433 cm^−1^ can be attributed to the O–H stretching of the hydroxyl groups associated with crystallized water or present on the surface of the SiO_2_ unit [30]. Additionally, M2 displays a small peak at 1634 cm^−1^, which is likely indicative of C=O stretching associated with organic residues [30].

The peak observed at 1423 cm^−1^ is indicative of C–C stretching associated with organic residues. Following this, the peak at 1083 cm^−1^ corresponds to the anti-symmetric stretching vibrations of the SO_4_^2−^ from CaSO_4_ in M2. The subsequent peaks at 1006 cm^−1^ and 776 cm^−1^ likely represent anti-symmetric and symmetric Si–O stretching vibrations, respectively [31]. Additionally, M2 exhibits peaks at 690 cm^−1^, 516 cm^−1^, and 459 cm^−1^, which are associated with the in-plane bending vibrations of C–O, Ca–O, and Al–O bonds, respectively.

**Figure 16:**
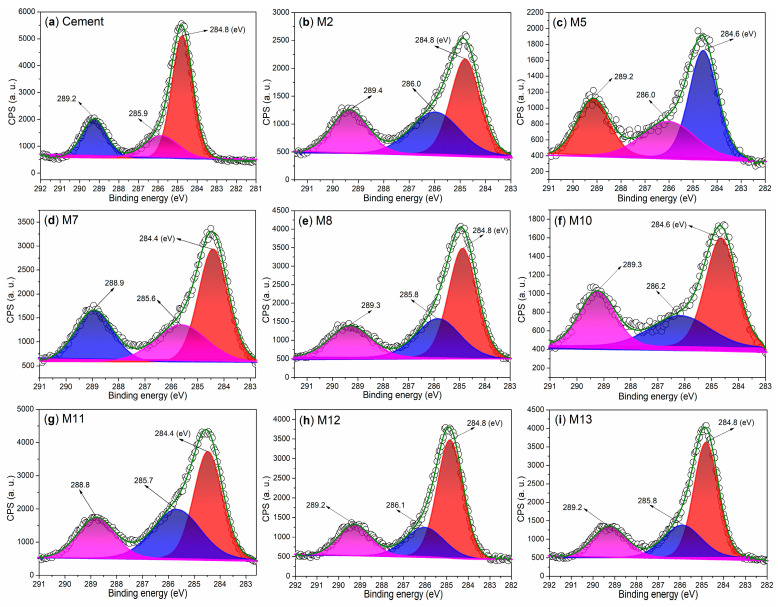
XPS measurements of C 1s regions: (**a**) cement, (**b**) M2, (**c**) M5, (**d**) M7, (**e**) M8, (**f**) M10, (**g**) M11, (**h**) M12, (**i**) M13; all mortars were tested at 24 h.

Other mortars, including M5, M8, M10, M12, and M13, exhibit FT-IR spectra that are similar to that of M2 (Figure 15b–f vs. Figure 15a), suggesting that they share comparable inorganic skeletons and organic species. From another perspective, the C 1s regions of cement and mortars generally reveal three distinct components (Figure 16a–i). The first component, appearing at 284.4–284.8 eV (Figure 16a–i), can be attributed to saturated carbons (sp^3^ hybridization). The subsequent components, found at 285.6–286.2 eV (Figure 16a–i), correspond to the carbons involved in C–O bonds, while the final components, located at 288.8–289.4 eV (Figure 16a–i), indicate the presence of carbons from carboxyl groups [36].

### 3.7. Proposed Processes for the Formation of Mortar Components During Cement Hydration

**Figure 17:**
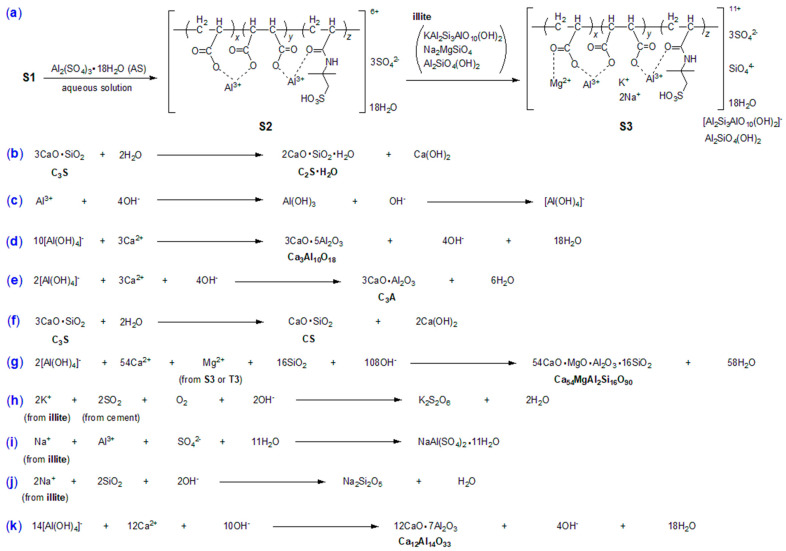
(**a**–**k**) The proposed processes for the formations of various mortar components during cement hydration facilitated by illite.

Based on the experimental results obtained thus far, the cement hydration processes facilitated by various admixtures are illustrated in Figure 17. Initially, the composition of S3 includes coordinated Al^3+^, Mg^2+^, K^+^, Na^+^, whose positive charges are balanced by SO_4_^2−^, SiO_4_^4−^, and other anions derived from illite (Figure 17a). Both cations and anions contribute to the subsequent transformations.

Next, the formation of C_2_SH in M2 (Figure 8b), M5 (Figure 8c), M7 (Figure 8d), M10 (Figure 8f), and M11 (Figure 8g) is depicted in Figure 17b, where the hydration of C_3_S in cement leads to the production of C_2_SH and Ca(OH)_2_, which is present in M12 (Figure 8h).

Furthermore, Figure 17c–f illustrate the formation processes of Ca_3_Al_10_O_18_ (M5, Figure 8c), C_3_A, and CS (M7, Figure 8d). The presence of Ca_54_MgAl_2_Si_16_O_90_ in M8 (Figure 8e), M12 (Figure 8h), and M13 (Figure 8i) can be explained by Figure 17g, which shows that the coated illite provides Mg^2+^. The formation of K_2_S_2_O_6_ observed in M8 (Figure 8e) is depicted in Figure 17h, where the K^+^ released from coated illite and the SO_2_ fixed in cement serve as the raw materials.

Moreover, NaAl(SO_4_)_2_·11H_2_O, identified in M10 (Figure 8f) and M11 (Figure 8g), is formed through the precipitation of Na^+^ (from coated illite), Al^3+^ (from coated AS), SO_4_^2−^, and H_2_O, as illustrated in Figure 17i. Another sodium salt, Na_2_Si_2_O_5_, present in M11 (Figure 8g), results from the combination of Na^+^ (from coated illite), SiO_2_, and OH^−^ (Figure 17j). Additionally, mayenite (Ca_12_Al_14_O_33_), found in M13 (Figure 8i), is expected to form as depicted in the transformation shown in Figure 17k.

## 4. Conclusions

In this study, two ternary hydrophilic copolymers were synthesized via ammonium persulfate-catalyzed free radical polymerization in an aqueous solution. These copolymers were then mixed with aluminum sulfate and ball-milled illite powder, resulting in two series of admixtures that were subsequently utilized in the cementing of Ordinary Portland Cement. The following conclusions can be drawn from this work:

1. The combination of aluminum sulfate with the synthesized copolymers acts as an active admixture in cementing, leading to reduced setting times and improved compressive and flexural strengths compared to those achieved with pure aluminum sulfate as an admixture. This enhancement is primarily attributed to the formation of adhesive pozzolanic clinkers, including Ca_3_Al_10_O_18_ (3CaO·5Al_2_O_3_), Ca_3_Al_2_O_6_ (C_3_A, 3CaO·Al_2_O_3_), NaAl(SO_4_)_2_·11H_2_O, and Na_2_Si_2_O_5_.
2. The illite-based admixtures, which consist of aluminum sulfate, copolymers, and ball-milled illite powder, exhibit slightly prolonged setting times, comparable or improved compressive strengths, and enhanced flexural strengths when compared to the admixtures made solely of aluminum sulfate and copolymers.
3. The illite-based admixtures demonstrate SiO_2_ as a skeleton material, rather than the Ca_2_SiO_4_·H_2_O (C_2_SH, 2CaO·SiO_2_·H_2_O) skeleton provided by the aluminum sulfate and copolymer admixtures. Furthermore, Ca_54_MgAl_2_Si_16_O_90_ (54CaO·MgO·Al_2_O_3_·16SiO_2_), a unique adhesive pozzolanic clinker, emerges in the presence of illite-based admixtures, significantly contributing to the mechanical strength development of the hydrated mortars (compressive strength at 24 h: M8 vs. M5, increased by 0.68%, M12 vs. M10, decreased by 1.93%; compressive strength at 28 d: M12 vs. M10, increased by 3.60%; flexural strength at 24 h: M8 vs. M5, increased by 31.10%, M12 vs. M10, increased by 108.21%).
4. The admixtures derived from the synthesized copolymer *p*(AA–*co*–IA–*co*–AMPS) generally demonstrate higher compressive strengths and comparable flexural strengths in mortars when compared to those derived from *p*(AA–*co*–MA–*co*–AMPS). This enhancement is primarily attributed to the extended carboxyl group linked by a methylene group on the IA (itaconic acid) monomer, which is likely to coordinate more tightly with Al^3+^ ions compared to the convergent carboxyl groups of the MA (maleic acid) monomer.
5. Microstructural, morphological, and thermal analyses of the hydrated mortars, along with discussions on the hydration processes, reveal that while illite itself is hydrophobic, the coating of ball-milled illite powder with aluminum sulfate and copolymers facilitates the dispersion of illite into the gaps and pores of the cement network during cementing. This process substantially increases the flexural strength of the hydrated mortars.

This study suggests that the integration of hydrophilic copolymers with mechanically activated clays can enhance the mechanical strength of hydrated mortars by effectively filling the pores and voids within the cement matrix. Importantly, this enhancement does not largely hinder the cementing process, which holds great potential for the future development of active cement admixtures.

## Figures and Tables

**Table 1:** The bromine numbers (*X*) and monomer conversions (*α*) of copolymers S1 and T1 ^a^.

Sample	*m* (g) ^b^	*V* (mL) ^c^	*X* (mg g^−1^) ^d^	*α* (%) ^e^
blank	-	20.68	-	-
S1	0.50	16.90	60.40	97.60
T1	0.50	16.30	69.99	96.92

^a^ As in Section 2.4. ^b^ Mass of tested sample. ^c^ Consumed volume of Na_2_S_2_O_3_ solution in titration. ^d^ Bromine number, determined by Equation (1). ^e^ Monomer conversion, determined by Equation (2).

**Table 2:** Binding energy and atomic composition of illite surface (depth, 0–3 nm).

O (1s)	C (1s)	Si (2p)	Al (2p)	K (2p)	Na (1s)	Mg (1s)
531.80 (45.40) ^a^	284.80 (8.07)	102.80 (24.91)	74.80 (20.69)	292.80 (0.79)	1070.80 (0.04)	1304.80 (0.10)

^a^ Binding energy (eV), along with atomic percentage (at%) in parentheses.

**Table 4:** Setting times of cement pastes facilitated by different admixtures ^a^.

Paste	Admixture (Dosage) ^b^	Setting Time (min)	
		Initial (IST)	Final (FST)
P1	blank	30.50 ± 0.45 ^c^	41.21 ± 0.77
P2	AS (7 wt.%)	19.11 ± 0.39	37.05 ± 0.69
P3	illite (7 wt.%)	25.55 ± 0.99	38.73 ± 0.32
P4	S1 (7 wt.%)	23.75 ± 1.57	36.44 ± 1.02
P5	S2 (7 wt.%)	2.44 ± 0.11	3.14 ± 0.15
P6	S2 (6 wt.%)	3.09 ± 0.21	4.76 ± 0.16
P7	S2 (8 wt.%)	2.04 ± 0.08	2.32 ± 0.05
P8	S3 (7 wt.%)	2.99 ± 0.13	4.35 ± 0.21
P9	S3 (8 wt.%)	2.69 ± 0.15	4.10 ± 0.12
P10	T2 (7 wt.%)	1.16 ± 0.05	2.02 ± 0.14
P11	T2 (8 wt.%)	1.10 ± 0.03	1.98 ± 0.09
P12	T3 (7 wt.%)	2.35 ± 0.10	3.98 ± 0.09
P13	T3 (8 wt.%)	3.25 ± 0.17	4.37 ± 0.21
P14	T3 (6 wt.%)	4.56 ± 0.26	5.96 ± 0.51

^a^ Experimental process as in Section 2.5. ^b^ Dosage (mass percentage) of admixture over cement, as in Section 2.5. ^c^ Data format: average value ± SD (standard deviation); for each paste, both average value and SD were obtained from three parallel experiments.

**Table 5:** Mechanical strengths of cement mortars facilitated by different admixtures ^a^.

Mortar	Admixture (Dosage) ^b^	Compressive Strength (MPa, Mortar)			Flexural Strength (MPa, Mortar)		
		6 h	24 h	28 d	6 h	24 h	28 d
M1	blank	0.91 ± 0.15 ^c^	4.10 ± 0.19	21.07 ± 0.56	0.20 ± 0.09	2.21 ± 0.12	12.29 ± 0.43
M2	AS (7 wt.%)	1.05 ± 0.06	5.51 ± 0.32	23.11 ± 1.10	0.71 ± 0.02	2.56 ± 0.19	11.17 ± 0.55
M3	illite (7 wt.%)	0.69 ± 0.20	3.70 ± 0.32	19.43 ± 0.16	0.21 ± 0.12	2.91 ± 0.32	12.30 ± 0.37
M4	S1 (7 wt.%)	0.98 ± 0.07	4.77 ± 0.14	22.33 ± 0.36	0.59 ± 0.13	2.76 ± 0.16	12.00 ± 0.15
M5	S2 (7 wt.%)	1.39 ± 0.10	5.81 ± 0.46	27.40 ± 1.01	0.91 ± 0.07	2.99 ± 0.05	13.15 ± 0.23
M6	S2 (6 wt.%)	1.12 ± 0.03	4.93 ± 0.22	23.95 ± 0.83	0.80 ± 0.01	2.10 ± 0.06	12.20 ± 0.17
M7	S2 (8 wt.%)	1.29 ± 0.08	5.26 ± 0.10	25.65 ± 0.36	1.11 ± 0.05	2.50 ± 0.16	12.88 ± 0.21
M8	S3 (7 wt.%)	1.70 ± 0.12	5.85 ± 0.14	28.31 ± 1.09	1.65 ± 0.17	3.92 ± 0.69	14.70 ± 0.19
M9	S3 (8 wt.%)	1.56 ± 0.07	5.14 ± 0.14	26.13 ± 1.02	1.36 ± 0.06	3.07 ± 0.17	13.99 ± 0.14
M10	T2 (7 wt.%)	2.24 ± 0.15	7.25 ± 0.18	29.97 ± 1.57	0.77 ± 0.09	2.80 ± 0.15	13.06 ± 0.13
M11	T2 (8 wt.%)	1.44 ± 0.25	5.44 ± 0.20	27.77 ± 0.96	0.80 ± 0.13	3.01 ± 0.06	14.38 ± 0.35
M12	T3 (7 wt.%)	2.19 ± 0.12	7.11 ± 0.08	31.05 ± 1.06	2.85 ± 0.22	5.83 ± 0.14	18.41 ± 0.91
M13	T3 (8 wt.%)	1.89 ± 0.06	6.74 ± 0.02	29.99 ± 0.74	3.01 ± 0.05	6.39 ± 0.13	21.03 ± 1.02

^a^ Experimental process as in Section 2.6. ^b^ Dosage (mass percentage) of admixture over cement, as in Section 2.6. ^c^ Data format: average value ± SD (standard deviation); for each mortar, both average value and SD were obtained from three parallel experiments.

**Table 6:** Compressive strength retention ratios of cement mortars after 28 days.

Mortar ^a^	*R*_28_ (%) ^b^
M5	130
M8	134
M10	142
M12	147

^a^ Corresponding to entries in Table 5. ^b^ Retention ratio after 28 days; *R*_28_ = *f*_t,28_/*f*_r,28_ × 100%; *f*_t,28_ means comprehensive strength of tested mortar at 28 d (MPa, admixture-facilitated); *f*_r,28_ means comprehensive strength of standard mortar (MPa, M1, Table 5, admixture-blank) at 28 d, according to Chinese standard GB/T 35159-2017 [40].

**Table 7:** Chemical composition of cement ^a^.

Composition	CaO	SiO_2_	Al_2_O_3_	Fe_2_O_3_	SO_3_	MgO	K_2_O	Na_2_O	Ignition Loss ^b^
Content (wt.%)	61.20	18.73	5.90	4.32	3.40	2.01	1.02	0.39	3.03

^a^ Chemical composition of cement (raw material), determined by ICP–OES. ^b^ Ignition loss, detected according to GB/T 34231-2017 [43].

**Table 8:** Binding energies and atomic compositions on surfaces of cement and mortars (depth, 0–3 nm; all mortars were tested at 24 h).

Sample ^a^	C (1s)	O (1s)	S (2p)	Si (2p)	K (2p)	Na (1s)	Ca (2p)	Al (2p)
Cement ^b^	284.80 (39.58) ^c^	531.80 (30.78)	168.80 (2.24)	101.80 (6.47)	293.5 (9.97)	1070.80 (0.09)	346.80 (10.72)	73.80 (0.15)
M2	284.80 (28.13)	530.80 (40.70)	168.80 (1.53)	101.80 (6.46)	293.5 (7.14)	1070.80 (0.21)	346.80 (14.16)	73.80 (1.67)
M5	284.80 (29.39)	530.80 (39.89)	168.80 (1.56)	100.80 (7.28)	293.5 (7.43)	1070.80 (0.20)	346.80 (14.16)	73.80 (0.09)
M7	284.80 (29.58)	530.80 (39.06)	168.80 (1.52)	100.80 (6.50)	293.5 (7.41)	1070.80 (0.04)	346.80 (13.41)	72.80 (2.48)
M8	284.80 (34.25)	531.80 (34.80)	168.80 (3.01)	101.80 (6.41)	293.5 (8.66)	1070.80 (0.37)	346.80 (12.11)	73.80 (0.12)
M10	284.80 (27.64)	530.80 (38.57)	168.80 (2.35)	100.80 (7.89)	293.5 (6.97)	1070.80 (0.03)	346.80 (14.40)	72.80 (2.15)
M11	284.80 (41.29)	531.80 (32.16)	168.80 (0.47)	101.80 (5.63)	293.5 (10.41)	1070.80 (0.29)	346.80 (9.70)	72.80 (0.05)
M12	284.80 (32.66)	530.80 (35.19)	168.80 (3.73)	100.80 (7.00)	293.5 (8.23)	1070.80 (0.32)	346.80 (11.25)	72.80 (1.62)
M13	284.80 (33.23)	530.80 (34.56)	168.80 (3.47)	100.80 (6.29)	293.5 (8.38)	1070.80 (0.03)	346.80 (11.54)	73.80 (2.50)

^a^ Same mortars as shown in Table 5. ^b^ Raw material, as in Section 2.1. ^c^ Binding energy (eV), along with atomic percentage (at%) in parentheses.

**Table 9:** Elemental and component mass percentages of 3 mortars, as well as crystalline sizes of mortar components derived from XRD.

Mortar ^a^	Elemental Mass Percentage (wt.%) ^b^					*d*_XRD_ (nm) (Component Mass Percentage, wt.%) ^c^	
	Na	Si	S	Mg	Al	SiO_2_	Ca_54_MgAl_2_Si_16_O_90_ (54CaO·MgO·Al_2_O_3_·16SiO_2_)
M8	0.21	24.31	0.80	0.31	1.74	82 nm	50 nm (52 wt.%)
M12	0.25	18.44	0.89	0.40	2.26	102 nm	42 nm (68 wt.%)
M13	0.24	22.75	0.90	0.34	1.87	89 nm	57 nm (57 wt.%)

^a^ Mortars all hydrated for 24 h. ^b^ Determined by ICP–OES. ^c^ Crystalline sizes of illite components were determined by XRD according to Scherrer’s equation [37], using 1, 0, 1 (h, k, l) diffraction of SiO_2_ and 0, 0, 9 diffraction of Ca_54_MgAl_2_Si_16_O_90_ for M8 and M12 (Figure 8e,h), and 0, 0, 2 diffraction of SiO_2_ and 0, 0, 9 diffraction of Ca_54_MgAl_2_Si_16_O_90_ for M13 (Figure 8i).

## Data Availability

The original contributions presented in this study are included in the article.
